# Integrated multi-omics analysis reveals the molecular landscape and therapeutic strategies in Chinese cervical cancer patients

**DOI:** 10.1371/journal.pbio.3004029

**Published:** 2026-10-05

**Authors:** Tao Pan, Xucui Zhuang, Ya Zhang, Jiyu Guo, Si Li, Can Zhang, Lili Han, Fuxia Li, Yueying Gao, Jinyang Yu, Yongsheng Li, Chaoyang Sun

**Affiliations:** 1 Key Laboratory of Reproductive Health Diseases Research and Translation of Ministry of Education & Key Laboratory for Human Reproductive Medicine and Genetic Research of Hainan Province, The First Affiliated Hospital of Hainan Medical University, School of Intelligent Medicine And Technology (Big Data Research Center), Hainan Medical University, Haikou, China; 2 Department of Obstetrics and Gynecology, National Clinical Research Center for Obstetrics and Gynecology, Tongji Hospital, Tongji Medical College, Huazhong University of Science and Technology, Wuhan, China; 3 Key Laboratory of Cancer Invasion and Metastasis (Ministry of Education), Hubei Key Laboratory of Tumor Invasion and Metastasis, Tongji Hospital, Tongji Medical College, Huazhong University of Science and Technology, Wuhan, China; 4 School of Interdisciplinary Medicine and Engineering, Harbin Medical University, Harbin, China; 5 Department of Gynecology, People’s Hospital of Xinjiang Uygur Autonomous Region, Urumqi, China; 6 Department of Gynecology, First Affiliated Hospital, Shihezi University, Shihezi, China; The University of Sheffield, UNITED KINGDOM OF GREAT BRITAIN AND NORTHERN IRELAND

## Abstract

Genomic or transcriptomic sequencing guides the precision of cervical cancer (CC) diagnosis; however, the molecular profile of Asian patients remains poorly understood. We established a comprehensive multi-omics cohort comprising 112 tumor-normal adjacent tissue pairs from Chinese CC patients. Our integrated analysis revealed a mutational burden comparable to other populations, with three major mutational signatures primarily attributed to APOBEC-associated, age-associated, and DNA repair-associated biological categories. Multi-omics profiling elucidated the functional consequences of somatic mutations and copy number variations, identifying PIK3CA, EP300, and MPRIP as key driver events. We further uncovered both concordant and discordant activation patterns across cancer-related signaling pathways. Transcriptomic and proteomic analyses nominated cell cycle regulation, epithelial-mesenchymal transition, and LAMC2 as promising diagnostic and therapeutic targets. Comprehensive immune characterization enabled stratification of patients into three major immune phenotypes with varying microenvironmental profiles. This study provides valuable resources and novel insights into the molecular architecture of cervical cancer in the Chinese population, identifying potential biomarkers to guide precision detection and immunotherapy strategies.

## Abstract 2

While cervical cancer is a major cause of cancer mortality in women, the molecular basis of disease heterogeneity in Asian populations remains unclear. This study identifies key molecular pathways and biomarkers from multi-omics profiles of 112 Chinese patient samples to guide therapeutic strategies.

## Introduction

Cervical cancer (CC) remains one of the most common malignancies among women worldwide, with persistent human papillomavirus (HPV) infection representing the primary etiological factor. Although HPV vaccination and screening programs have reduced disease incidence in some regions, cervical cancer continues to cause substantial morbidity and mortality, particularly in low- and middle-income countries where access to preventive and therapeutic strategies remains limited [1]. Genomic, transcriptomic, epigenomic, and proteomic studies have therefore been essential for defining recurrent driver events, molecular subtypes, and therapeutically relevant vulnerabilities in this disease.

Comprehensive genomic studies have substantially advanced our understanding of cervical cancer biology. It has been reported that recurrent genomic alterations in cervical cancer frequently involve key driver genes such as PIK3CA, EP300, and FBXW7, together with broader dysregulation of chromatin remodeling and oncogenic signaling pathways [2]. Subsequently, The Cancer Genome Atlas (TCGA) study integrated genomic, transcriptomic, epigenomic, and proteomic datasets from cervical carcinomas and defined recurrent molecular alterations involving PI3K/MAPK signaling, immune-related pathways, and HPV-associated genomic remodeling [3]. These studies established the molecular landscape of cervical cancer; however, most patients included in these cohorts were of European or North American ancestry, and Asian populations remain underrepresented.

Beyond genomic alterations, recent multi-omics studies have highlighted the importance of transcriptional and microenvironmental heterogeneity in cervical cancer. Previous multi-omics analyses have shown that cervical cancer can be stratified into molecular subtypes with distinct EMT-related, Hippo/YAP-associated, and immune microenvironmental features [4]. These findings suggest that cervical cancer heterogeneity cannot be fully explained by genomic alterations alone and emphasize the importance of integrated multi-dimensional profiling.

Despite these advances, comprehensive multi-omics characterization of cervical cancer in Asian populations remains limited. Given potential differences in genetic background, HPV genotype distribution, environmental exposure, and immune landscape, whether previously defined molecular subtypes and immune phenotypes are applicable to Asian patients remains unclear. Therefore, large-scale multi-omics profiling of Asian cervical cancer cohorts is essential to define population-specific molecular characteristics and identify clinically actionable biomarkers.

Here, we performed integrated proteogenomic analyses—spanning genomics, transcriptomics, proteomics, and phosphoproteomics—on matched tumor and adjacent normal tissues from 112 Chinese cervical cancer patients. Our study emphasizes clinically actionable insights derived from proteomic and post-translational modification profiles, capturing functional consequences of genomic alterations. This approach revealed distinct disease subgroups with prognostically relevant phenotypes, driven by underlying genetic variations. Additionally, we characterized the tumor immune microenvironment, identifying potential predictive markers and therapeutic targets to guide immune checkpoint inhibitor (ICI) selection. Collectively, this work provides a comprehensive multi-omics resource that delineates the pathogenic mechanisms of CC and proposes candidate biomarkers, thereby supporting future efforts in precision diagnostics and targeted therapy development for this population.

## Results

### Proteogenomic characterization of CN-CC tumor and NAT samples

To comprehensively characterize the molecular landscape of Chinese cervical cancer (CN-CC), we curated high-quality tumor and normal adjacent tissue (NAT) samples from 112 patients (Figs 1A and S1A; S1 Table). The cohort included both squamous cell carcinomas and adenocarcinomas, with squamous cell carcinoma representing the predominant histological subtype ([*n* = 108, 96.4%] squamous cell carcinomas; [*n* = 4, 3.6%] adenocarcinomas). Whole-exome sequencing (WES), RNA sequencing (RNA-seq), isobaric tandem mass tag (TMT) labeling-based global proteomics, and phosphoproteomics were performed on the cohort samples.

**Fig 1:**
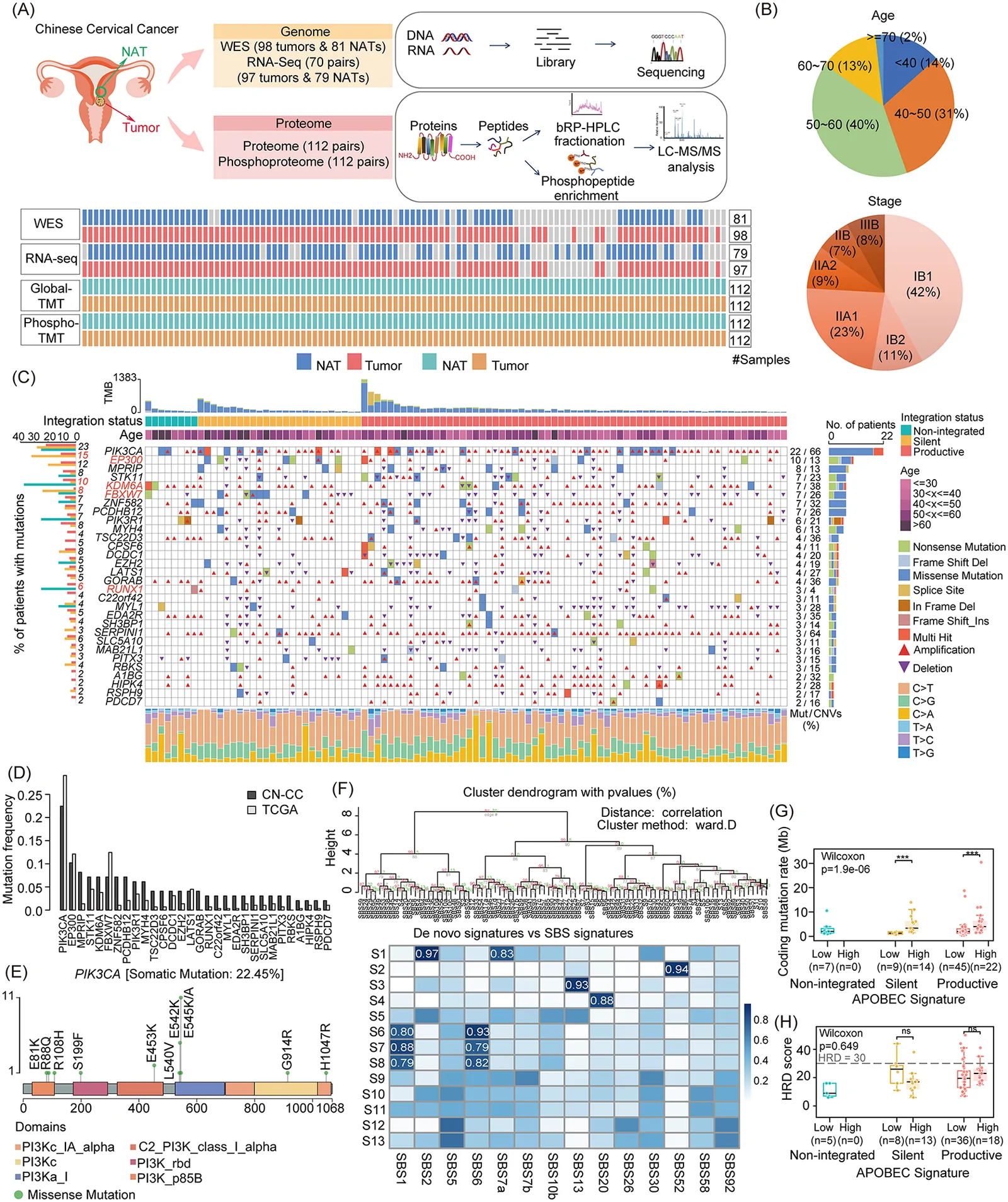
Multiomics landscape of CN-CC samples. **(A)** Overview of the experimental design and number of samples for proteomics, phosphoproteomics, WES, and RNA-seq analyses. The gray blocks in the schematic represent the missing data. The annotations to the right indicate samples in each category (bottom panel). WES, whole-exome sequencing. **(B)** Clinical information on the CN-CC samples. **(C)** Genetic profiles and associated clinicopathologic features of all 98 CC patients. **(D)** Comparisons of the top 30 significantly mutated genes between the CN-CC cohort and the TCGA CESC cohort. **(E)** Lollipop plot visualizing all protein-altering somatic mutations detected in this cohort. The size of a lollipop represents the number of samples with corresponding mutations, and the color represents a specific type of mutation. **(F)** Unsupervised clustering results between the COSMIC signatures and 13 signatures of the CN-CC cohort. **(G)** Differences in coding mutation rates across sample types exhibiting low (≤0.4) and high (>0.4) APOBEC signatures (*n* = 98 WES tumors). **(H)** Differences in coding HRD scores across sample types exhibiting low (≤0.4) and high (>0.4) APOBEC signatures (*n* = 81 paired tumor-normal WES samples). The data underlying the graphs shown in the figure can be found in S1 Data.

In total, we revealed 46,691 somatic mutations, including 13,212 missense, 1,364 nonsense, 5,927 silent, 1,022 splice site, 28 nonstop mutations, 292 frameshift insertions (ins) and deletions (indels), 81 in-frame ins and indels and 24,765 noncoding mutations in CN-CC cohort. We next analyzed the proteomics and phosphoproteomic data, which revealed 6,595 proteins and 17,824 phosphopeptides from 5,165 proteins, encompassing 17,824 securely localized phosphosites, with 15,605 (87.6%) being serine, 2,098 (11.8%) being threonine, and 121 (0.6%) being tyrosine.

We also collected the clinical parameters of the patients, including age, tumor grade and stage (Fig 1B and S1 Table). Over 70% of the enrolled samples were from patients aged 40–60 years, while the proportion of patients under 40 years exceeded 10%. Many patients in the CN-CC cohort are diagnosed at advanced stages, possibly because of factors such as inconspicuous early symptoms, lack of timely treatment, inappropriate treatment methods, poor treatment outcomes, and cancer cell metastasis (Fig 1B). The integration of HPV into the host genome is a critical event in the development and progression of CC. Thus, we conducted genomic analysis on paired tumors and adjacent tissues from 98 CC patients, including eight samples without HPV integration into the human genome (non-integrated), 25 samples with integrated HPV genomes but no transcriptional activity, indicating no virus RNA production (silent), and 65 samples with integrated HPV genomes undergoing transcription, resulting in active virus RNA production (productive) (Fig 1C).

### Genomic landscape of the CN-CC cohorts

We identified somatic mutations in 98 tumor-normal pairs through whole-exome sequencing analysis. MutSigCV detected significantly mutated genes (SMGs), including PIK3CA (22%), EP300 (10%), MPRIP (8%), and STK11 (7%) (Fig 1C). Mutation frequencies of EP300, KDM6A, FBXW7, and RUNX1 differed significantly across HPV subgroups (non-integrated, silent, productive; Fisher’s exact test, adjusted *p* < 0.05). Comparison with the TCGA cohort revealed similar mutation frequencies among the top 30 SMGs and a comparable tumor mutation burden (Figs 1D and S1B). Mutations in PIK3CA and EP300 have been previously implicated in cervical and other cancers [3]. PIK3CA mutations were predominantly localized to the helical domain (E542K, E545K), a pattern consistent with bladder cancer and HPV-positive head and neck squamous cell carcinomas, but distinct from breast cancer (Fig 1E). These substitutions are potentially linked to APOBEC-mediated mutagenesis [5]. EP300 mutations clustered predominantly within the zf-TAZ domain, which mediates transcriptional activation via interaction with other transcription factors (S1C Fig). Initial de novo extraction identified 13 mutational patterns across the cohort (S1D–S1G Fig). However, comparison with COSMIC SBS signatures revealed that several extracted signatures showed substantial overlap with known mutational processes (S2 Table). In particular, the S5 signature demonstrated limited similarity to SBS10b and lacked independent biological relevance, whereas its mutation spectrum closely resembled APOBEC-associated SBS2 and SBS13 (Fig 1F). Therefore, S5 was considered a composite APOBEC-related pattern rather than an independent mutational process. Mutational processes were consolidated into four major biological categories, including APOBEC-associated, age/background, DNA repair-associated, and low-confidence/artifactual signatures (S1H–S1J Fig).

Notably, the APOBEC-associated signature was significantly enriched in both silent and productive HPV-integrated tumors, suggesting HPV integration may contribute to APOBEC mutagenesis (S1H–S1J Fig). Tumors with high APOBEC signature burden (proportion > 0.4) exhibited a greater number of coding mutations—especially in silent and productive cervical cancers—consistent with prior reports [6]. Homologous recombination deficiency (HRD) was inferred computationally, revealing that APOBEC-high tumors also presented elevated HRD scores (Fig 1H). Ten samples (12.3%, 10/81) showed moderate-to-high HRD (>30), indicative of homologous recombination repair pathway dysregulation [6]. These findings suggest that APOBEC activity is linked to increased mutational load and elevated HRD, which may influence therapeutic vulnerability to agents such as PARP inhibitors in cervical cancer [7].

### Impact of genomic alterations on transcriptome, proteome, and phosphorylation

Based on analysis of somatic copy number variations (CNVs), the non-integrated cohort exhibited the highest genomic stability, whereas the silent cohort showed moderate stability. In contrast, the productive cohort harbored significantly fewer CNVs. Recurrent CNV patterns—including amplifications on chromosomes 1, 19, 20, X, and arm 3q, and deletions on chromosomes 11, 13, and arms 3p and 4q—were observed in both silent and productive cohorts (Fig 2A). The silent cohort displayed three significantly amplified chromosomal arms, while the productive cohort had three amplified and five deleted arms; the non-integrated group showed only two amplified arms. Differential gene expression analysis in CNV regions across HPV subtypes revealed distinct pathway enrichments: non-integrated samples were linked to RNA degradation and IL-17 signaling, whereas silent and productive samples were associated with oxidative phosphorylation, the tricarboxylic acid cycle, cell cycle, PI3K-AKT and TGF-β signaling, and other cancer-related pathways (Fig 2B and 2C).

**Fig 2:**
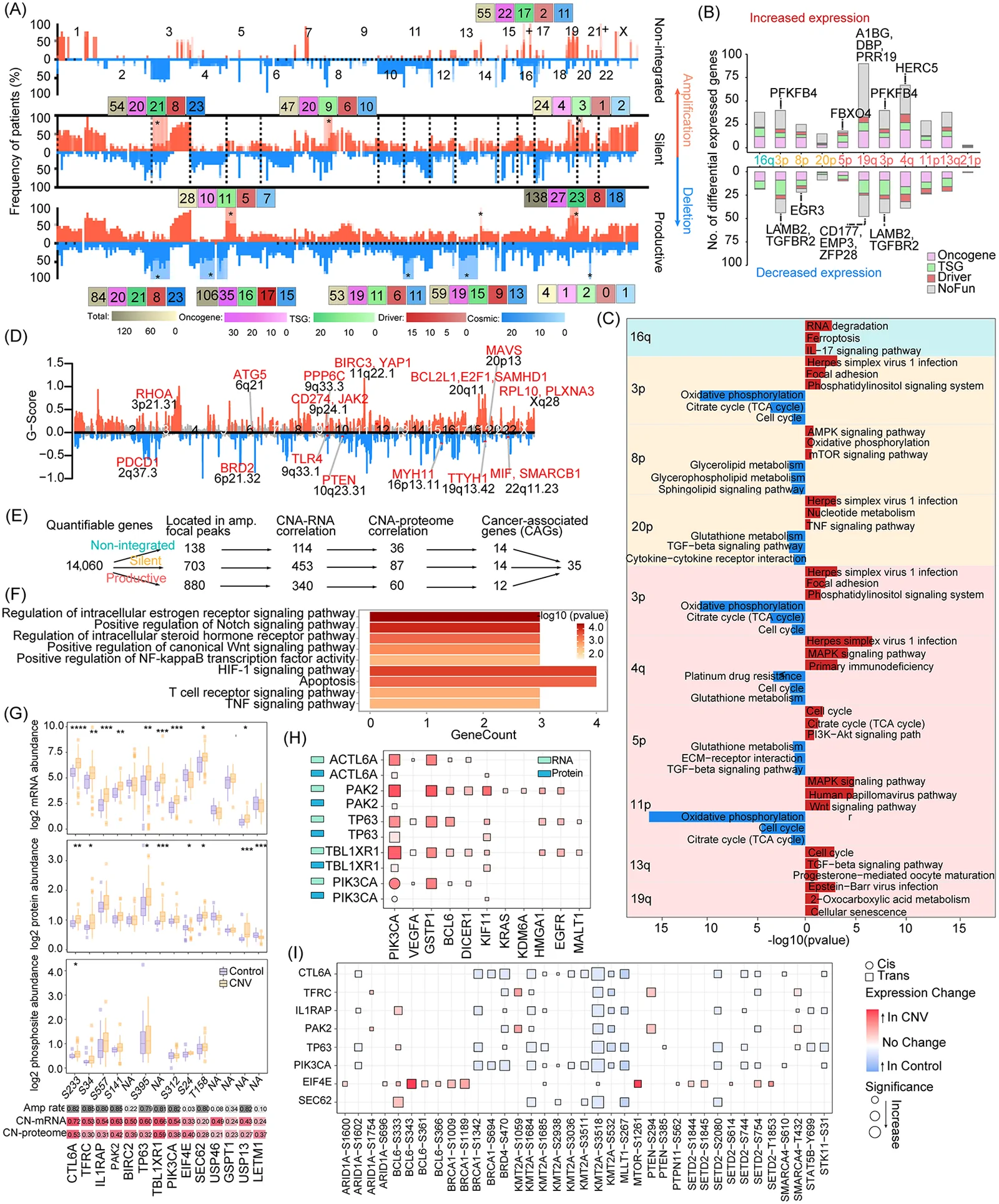
Impact of genomic alterations on the transcriptome, proteome, and phosphoproteome. **(A)** Broad somatic copy number alterations in our nonintegrated, silent and productive samples. An asterisk (*) indicates that the region is significantly amplified or deleted (as determined by GISTIC, *q* < 0.25) in only silent or productive samples, and a plus sign (+) indicates that the region is significantly amplified or deleted in only nonintegrated samples. The top number represents the number of genes in that region that are associated with cancers. **(B)** Differentially expressed genes in significantly amplified or deleted regions. **(C)** Significantly amplified or deleted regions of differentially expressed genes participating in functional pathways. **(D)** Effects of CNV on the mRNA and protein expression of CN-CC samples. Focal peaks with significant copy number gains (red) and losses (blue) (GISTIC2, *q* < 0.25) are shown. The cancer-related genes encoded from these focal peaks are highlighted in the CancerMine database. **(E)** Schematic of the CNV driver approach. Among all the genes with copy number events, 35 driver genes were located in the GISTIC focal peaks. **(F)** The enriched pathways of CNV driver genes. **(G)** Box plots showing the impact of copy number on a select number of proteins from 14 out of 35 putative CNV drivers. Wilcoxon rank-sum test, * *p* < 0.05, ** *p* < 0.01, *** *p* < 0.001, **** *p* < 0.0001. The control group in each comparison included all samples without copy number events for each gene, protein or phosphoprotein. **(H)** *Cis*- and *trans*-effects of CNV on RNA and protein levels. **(I)** *Cis*- and *trans*-effects of CNV on phosphosite levels. In **H** and **I**, the *cis* effects are denoted by circles, whereas the *trans* effects are denoted by squares. The data underlying the graphs shown in the figure can be found in S1 Data.

Significant localized amplifications (e.g., 3p21.31, 10q23.31, 20q13) and deletions (e.g., 2q37.3, 10q23.31, 16p13.11) were identified (Figs 2D and S2A–S2C), involving known cancer genes including PTEN, CD274, and YAP1 [3]. Gene and protein expression levels were generally upregulated in amplified regions and downregulated in deleted regions (S2D Fig), exemplified by PTEN loss activating the phosphoinositide 3-kinase (PI3K) β signaling to promote tumor survival [8]. Correlation analysis of CNV-mRNA/protein/phosphoprotein pairs revealed 5,588, 1,636, and 902 significant associations, respectively (S2E–S2G Fig), with *cis-*effects observed in 39.7%, 24.8%, and 17.5% of cases (S2H Fig). mRNA abundance showed a slightly stronger correlation with CNV (median *ρ* = 0.29) than protein abundance (median *ρ* = 0.26), consistent with prior reports [9]. Among 868 proteins exhibiting both CNV-mRNA and CNV-protein *cis-*effects, enriched pathways included MAPK signaling, T/B-cell receptor signaling, viral carcinogenesis, and TNF signaling (S2I Fig), underscoring links between cervical cancer and immune regulation.

Through correlation analysis, 35 cancer-related genes were prioritized (Fig 2E). These genes were enriched in pathways critical for tumorigenesis and immunity, such as intracellular estrogen receptor signaling, Notch, NF-κB, and T-cell receptor signaling (Fig 2F). Their mRNA, protein, and phosphoprotein abundances were generally elevated in cervical cancer (CC) (Fig 2G). While most genomic alterations led to concordant increases in RNA and protein levels but decreased phosphorylation (Fig 2H and 2I), TP63 alterations exhibited an inverse pattern between protein and phosphoprotein levels, possibly reflecting extensive post-translational regulation.

### Integrated multi-omics analyses of CN-CCs and NATs

To explore the differences between CCs and NATs, principal component analysis (PCA) was used to reveal significant differences at the transcriptome, proteome, and phosphoproteome levels (S3A–S3C Fig). The gene-protein expression correlation was significantly stronger in CCs (median = 0.195) than in NATs (median = 0.044; *p* < 2.2e−16, Wilcoxon rank-sum test; S3D Fig), consistent with other cancers [10]. Multi-omics integration identified genes perturbed in CCs (Figs 3A, S3E, and S3F). CC-upregulated genes were enriched in cancer-related pathways (e.g., viral infection, E2F/MYC targets, mTORC1), whereas NAT-upregulated genes were linked to metabolism (Figs 3B and S3E–S3K).

**Fig 3:**
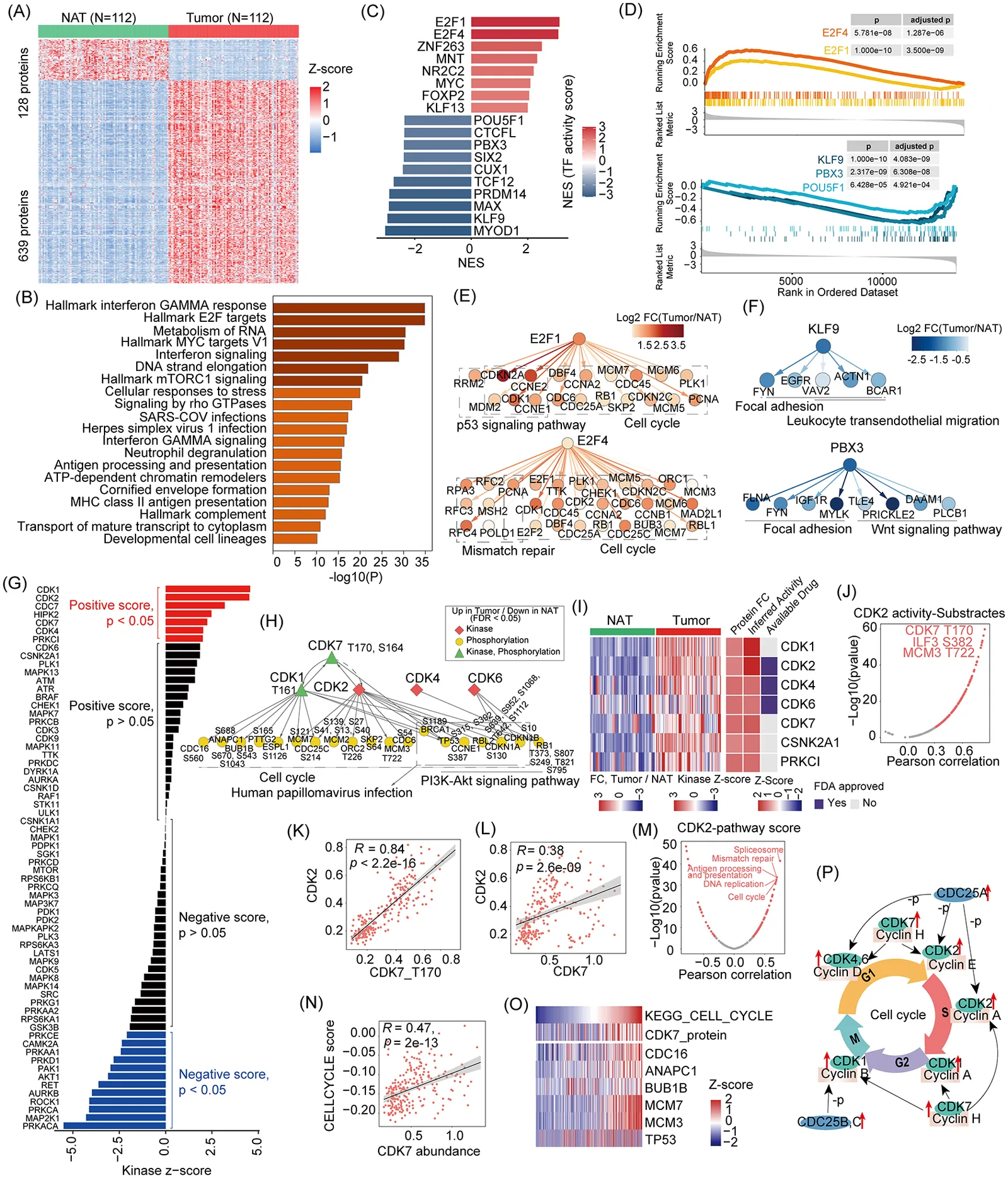
Integrated multiomics analyses of tumor tissues compared with NATs. **(A)** Differentially expressed protein profiles in tumor and NAT samples. **(B)** Enrichment analysis of significantly differentially expressed proteins in tumor samples. **(C)** Comparison of TF activity inference from transcriptomic data between tumors and NATs. **(D)** GSEA plots showing the TF activity of E2F1, E2F4, KLF9, PBX3 and POU5F1 in tumors and NATs. **(E, F)** TF and downstream target gene network diagram illustrating differences between tumors and NATs in terms of target abundance and key pathway regulation. **(G)** Evaluation of kinase activities by KSEA in tumors. **(H)** Kinase-substrate network diagram illustrating the differences between tumors and NATs in terms of kinase activity and corresponding phosphorylated substrate abundance. **(I)** Heatmap showing the mRNA expression levels of activated kinases in tumors and NATs. Inferred activity was calculated via KSEA, and purple boxes indicate the existence of an FDA-approved drug. **(J)** Volcano plot showing the downstream phosphosites for CDK2 kinase activity. **(K)** Spearman correlation between kinase substrate CDK7-T170 phosphorylation site abundance and CDK2 protein abundance. Spearman correlation *rho* = 0.84, *p* < 2.2e − 16. **(L)** Spearman correlation between the protein abundance of the kinase substrate CDK7 and the protein abundance of CDK2. Spearman correlation *rho* = 0.38, *p* < 2.2e−16. **(M)** Spearman correlation between CDK2 protein abundance and pathway score. **(N)** Spearman correlation between CDK2 protein abundance and the pathway score of the cell cycle. Spearman correlation *rho* = 0.47, *p* < 2e−13. **(O)** Heatmap of the protein abundance of cell cycle-related genes. **(P)** Schematic diagram of the cell cycle in CN-CC patients. The data underlying the graphs shown in the figure can be found in S1 Data.

Comparative analysis of transcription factor (TF) activity revealed significant activation of eight TFs (including E2F1, E2F4, MYC, MNT) in CCs (NES > 0, *p* < 0.05), while POU5F1, PBX3, and KLF9 were downregulated (Fig 3C and 3D; S3 Table). Target gene analysis confirmed elevated expression of E2F1/E2F4-regulated genes involved in p53 signaling, mismatch repair, and cell cycle pathways (Figs 3E and S3K). Conversely, KLF9/PBX3 targets associated with focal adhesion, WNT signaling, and leukocyte transendothelial migration showed reduced expression (Figs 3F and S3L).

Aberrant regulation of protein kinases may be implicated in diverse oncogenic processes [11]. Kinase-substrate enrichment analysis (KSEA) identified seven kinases, including CDK1, CDK2, CDC7, and CDK7, as significantly activated in cervical carcinomas (CCs) (|*m*| > 4, *Z* score > 2; Fig 3G and S4 Table). Network analysis highlighted CDK involvement in cell cycle regulation, HPV infection, and PI3K-AKT signaling (Fig 3H). Activated kinases, including CDK2, CDK4, and CDK6—which are targeted by FDA-approved inhibitors—were significantly upregulated (Fig 3I), aligning with the PKIDB database [12].

Phosphoproteomic correlation analysis revealed that CDK2 activity was most strongly associated with CDK7 phosphorylation at T170 (Fig 3J), and CDK2 abundance correlated with CDK7 levels (Fig 3K). CDK2 abundance further correlated with the activity of cell cycle, DNA replication, and mismatch repair pathways (Fig 3L). Notably, CDK2-mediated regulation of CDK7 protein levels was linked to cell cycle signaling activity (Fig 3M). Consistent with this, elevated CDK7 abundance correlated with increased levels of cell-cycle-related proteins (e.g., CDC16, MCM7, TP53; Fig 3N and 3O), collectively delineating an aberrant cell cycle regulatory network in CCs (Fig 3P).

### HPV integration status-associated proteogenomic alterations in CN-CC

We performed integrated proteomic and phosphoproteomic profiling of 112 paired cervical cancer samples, quantifying 6,595 proteins and 17,824 phosphosites (15,605 pSer, 2,098 pThr, 121 pTyr). To determine whether HPV integration status was associated with coordinated molecular alterations, we performed supervised comparisons of transcriptomic, proteomic, and phosphoproteomic profiles across non-integrated, silent, and productive tumors. The supervised heatmaps revealed distinct HPV integration status-associated molecular patterns across all three omics layers included oncogenes, tumor suppressors, and tissue-enhanced genes (THE), with enrichment in key tumorigenic pathways (Figs 4A and S5) [13]. These patterns indicate that HPV integration status is a major determinant of the observed proteogenomic variation, rather than supporting three molecular subtypes independent of HPV integration status.

**Fig 4:**
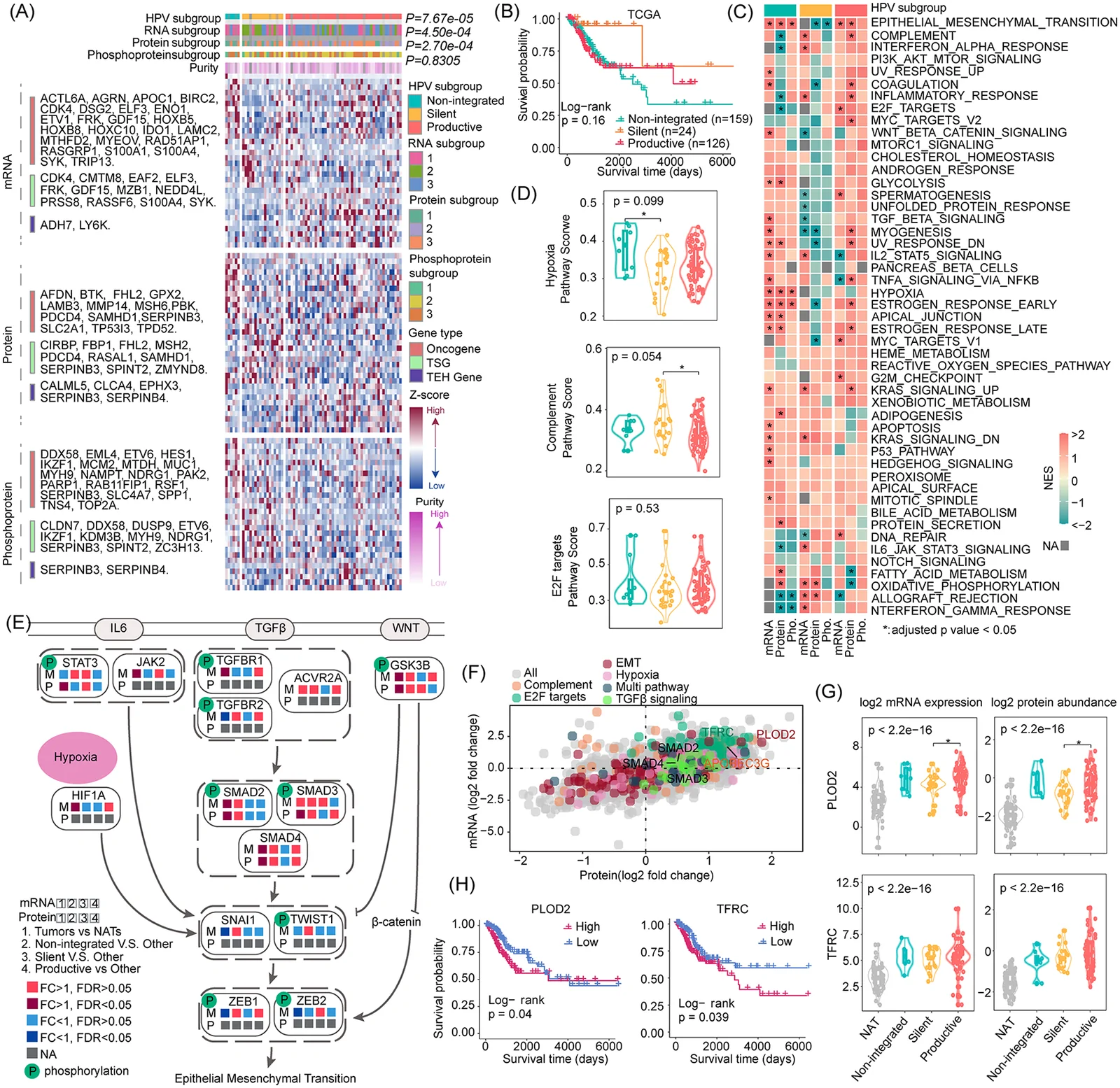
Comparative proteogenomic characteristics of cervical cancer subgroups. **(A)** Supervised proteogenomic heatmaps of cervical tumors grouped by HPV integration status. Samples are ordered as non-integrated, silent, and productive tumors. The heatmaps show differentially expressed transcripts, proteins, and phosphosites among the three HPV-defined groups after row-wise Z-score normalization. Top annotations indicate HPV integration status, tumor purity, and relevant clinicopathological features. The *p* values for categorical variables were calculated via the chi-squared test. **(B)** Kaplan–Meier curves for overall survival based on RNA expression in the TCGA project cohort. *n* = 2,309, log-rank test. **(C)** Integrated analysis of differentially activated metabolic and signaling pathways at the mRNA, protein, and phosphoprotein levels among the three CC subgroups. **(D)** Pathway scores of hypoxia, complement and E2F_target signaling pathways between subtypes. The line and box represent the median and upper and lower quartiles, respectively. **(E)** Diagram showing the multiomics profiles of coregulators of the EMT signaling pathway. **(F)** Identification of key regulatory factors in CC-related signaling pathways. Scatter plots showing the log2-fold change in the proteome vs. mRNA between tumors and paired NATs. Different colored dots represent key regulatory factors involved in different signaling pathways. **(G)** Boxplot illustrating PLOD2 and TFRC mRNA and protein expression between subtypes as well as across proteomic subgroups. **(H)** Survival Kaplan–Meier curves according to the PLOD2 and TFRC gene expression levels. The data underlying the graphs shown in the figure can be found in S1 Data.

We investigated survival outcomes based on HPV integration status using TCGA clinical data, revealing that samples with productive HPV integration exhibited poorer prognosis compared to silent samples (Fig 4B). Multi-omics pathway enrichment analysis demonstrated that nonintegrated samples were enriched in epithelial-mesenchymal transition (EMT), hypoxia, and early estrogen response pathways (Fig 4C). Silent samples showed enrichment in allograft rejection and interferon response pathways, while productive samples were linked to EMT, MYC targets, E2F targets, and DNA repair. Correspondingly, pathway activity scores highlighted elevated hypoxia in nonintegrated samples, increased complement in silent samples, and higher E2F target and hypoxia activity in productive samples (Fig 4D). Multi-omic profiling further revealed dysregulation of key EMT pathway components, including increased HIF1A and SMAD family expression, decreased ZEB1/ZEB2, and altered phosphorylation patterns (Fig 4E).

Proteogenomic analysis identified key regulatory factors in cervical cancer signaling pathways. Multi-omics profiling revealed coordinated upregulation of E2F target pathway components at both transcriptional and translational levels (Fig 4F). Notably, transferrin receptor (TFRC) and procollagen-lysine, 2-oxoglutarate 5-dioxygenase 2 (PLOD2), both implicated in E2F signaling, were significantly overexpressed across omics layers in cervical tumors (Fig 4G). Survival analysis in the TCGA cohort showed only modest and time-dependent differences in overall survival according to TFRC and PLOD2 expression levels (Fig 4H). These findings delineate subtype-specific molecular signatures and nominate potential therapeutic targets for cervical cancer intervention.

### Identification of diagnostic and therapeutic biomarkers

Integrated multi-omics analysis nominated 12 candidate tissue-associated proteins that were differentially expressed between cervical tumors and NATs and may reflect tumor-associated biological programs (Fig 5A). Consistent with prior evidence linking elevated PARP1 mRNA to poor prognosis [14], we observed significant upregulation of STAT1 and PARP1 at both mRNA and protein levels (S6A and S6B Fig). LAMC2 protein abundance was markedly elevated in tumors, with highest expression in nonintegrated subtypes (Fig 5B). Similarly, IDO1 was upregulated at transcriptomic and proteomic levels, showing distinct expression patterns among integration subtypes: lowest in nonintegrated, higher in productive, and highest in silent samples (Fig 5C). These proteins represent promising molecular targets for subtype-specific intervention.

**Fig 5:**
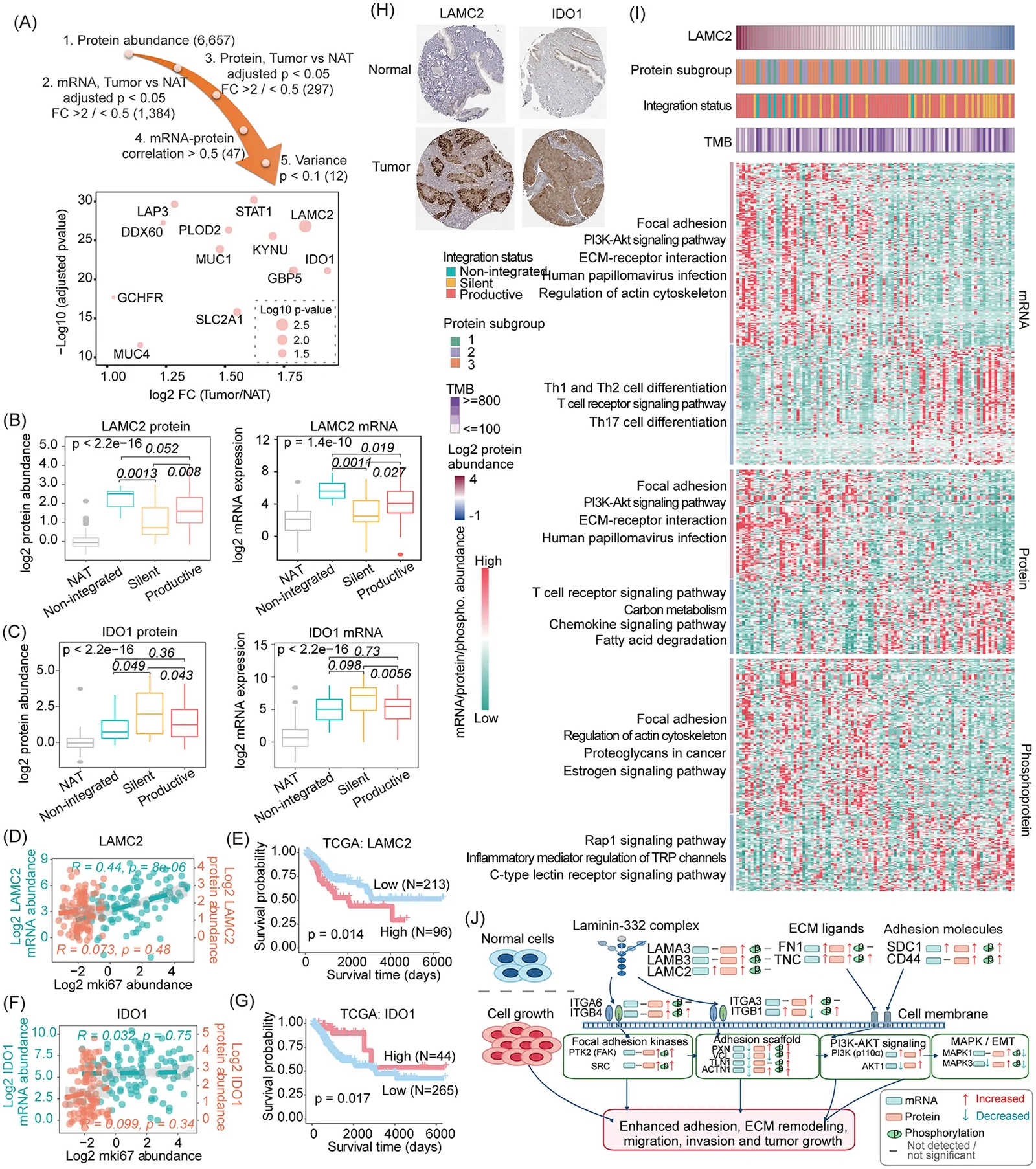
Proteomic detection and therapeutic biomarkers. **(A)** Workflow for selecting key regulatory proteins, with dots showing the 12 candidate proteins. FC, fold change. **(B)** Relative protein abundance and mRNA expression of LAMC2 between tumors and NATs as well as across cervical cancer subgroups. The line and box represent the median and upper and lower quartiles, respectively. **(C)** Relative protein abundance and mRNA expression of IDO1 between tumors and NATs as well as across cervical cancer subgroups. The line and box represent the median and upper and lower quartiles, respectively. **(D)** Spearman correlation between gene expression or protein abundance of LAMC2 and MKI67 in cervical cancer. **(E)** Kaplan–Meier curves for overall survival based on the mRNA expression of LAMC2 in an independent TCGA CESC cohort. *n* = 309, log-rank test. **(F)** Spearman correlation between gene expression or protein abundance of IDO1 and MKI67 in cervical cancer. **(G)** Kaplan–Meier curves for overall survival based on the mRNA expression of IDO1 in an independent TCGA CESC cohort. *n* = 309, log-rank test. **(H)** Representative images of LAMC2 and IDO1 immunostaining in tumor and nontumor cervical tissues from the HPA. **(I)** Association of LAMC2 expression with proteomic subgroups, clinicopathologic factors, and multiomics profiles. **(J)** Multi-omics model of LAMC2-associated ECM-receptor interaction in cervical cancer. The data underlying the graphs shown in the figure can be found in S1 Data.

Multi-omics analysis confirmed that MKI67 was consistently upregulated at both transcriptomic and proteomic levels in cervical cancer, with expression progressively increasing across HPV integration stages (S6C Fig). Candidate biomarkers, including STAT1, PARP1, LAMC2, and IDO1, showed significant positive correlations with MKI67 expression (S6D Fig), with LAMC2 exhibiting a particularly strong transcriptional correlation (Fig 5D). Survival analysis in an independent TCGA cohort and high protein expression confirmed in the HPA database underscore the prognostic relevance of LAMC2 and IDO1 (Fig 5H). Stratification by LAMC2 expression revealed significant multi-omics alterations in the high-expression group, with upregulated genes enriched in key oncogenic pathways such as focal adhesion, PI3K-AKT, HPV infection, and ECM-receptor interaction (Fig 5I). These findings emphasizes a LAMC2-associated ECM-receptor interaction model involving laminin-332 components, integrin receptor axes, focal adhesion kinases/scaffold proteins, and downstream PI3K-AKT and MAPK/EMT-related signaling outputs (Fig 5J).

### Characterization of immune cell infiltration in the CN-CC cohort

We characterized the immune infiltration landscape of cervical cancer using xCell deconvolution and ESTIMATE-based immune scoring. Based on the overall immune and stromal infiltration patterns, tumors were classified into three major immune phenotypes: immune-hot, immune-warm, and immune-cold (Fig 6A) [15]. The immune-hot phenotype showed higher immune infiltration, whereas the immune-cold phenotype showed markedly reduced immune and stromal signals, with the immune-warm phenotype displaying intermediate features. This three-phenotype framework was used for the main downstream analyses because it provided the clearest biological and clinical interpretation of the immune landscape (Figs 6A, 6B, and S7A–S7D). The immune-hot tumors exhibited elevated infiltration scores, particularly of effector memory CD4^+^ T cells, macrophages, and NKT cells. Crucially, this phenotype showed significant upregulation of key immune checkpoint mediators (CTLA4, PD-1 (PDCD1), PD-L1 (CD274), IDO1, LAG3) and cytotoxic factors (GZMA, GZMB, PRF1) at both transcriptomic and proteomic levels. Elevated expression of immune evasion factors (CD40, WARS, SAMD9L, GBP1, GBP5) and T-cell exhaustion markers (CD274, CD8A, CTLA4) was also observed (Fig 6A). Strong correlations between ESTIMATE immune scores and immune-related gene expression further validate this stratification. These findings highlight the immune-hot subtype as a prime candidate for ICI therapy.

**Fig 6:**
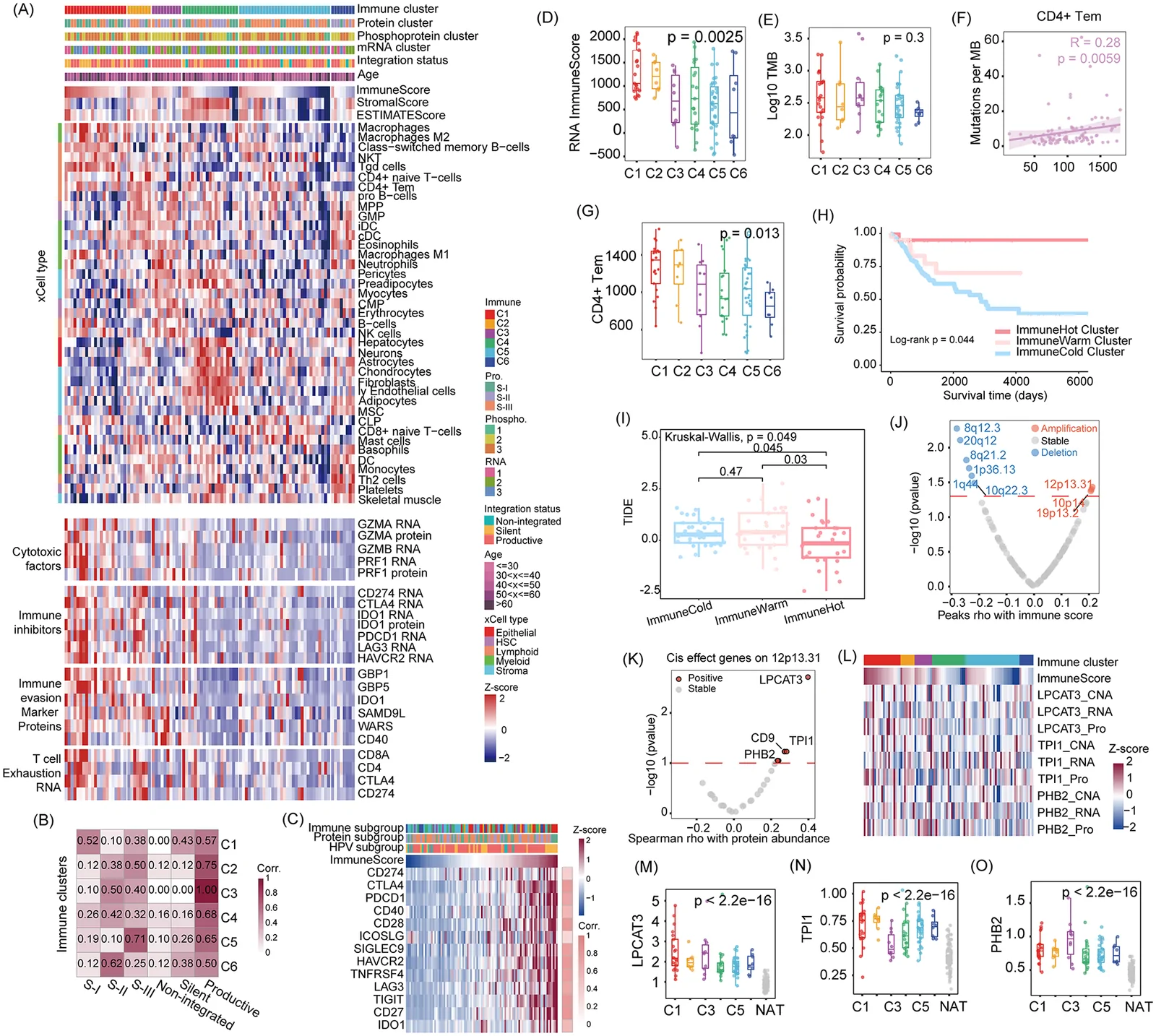
Immune landscape of the CN-CC cohort. **(A)** Heatmap illustrating the immune infiltration features and activity profiles of significant pathways across the six immune clusters. The top panel shows the immune subtypes, selected significant clinical features, transcriptomic subtypes, proteomic subtypes and phosphoproteomic subtypes. The second graph panel shows deconvoluted immune/stromal and ESTIMATE signatures via the xCell and ESTIMATE algorithms on the basis of transcriptomic data, together with the derived relative abundances of immune and stromal cell types. The third graph shows the mRNA and protein abundances of key immune-related markers. **(B)** Heatmap illustrating the overlap of immune clusters with proteomic subtypes and HPV subgroups. **(C)** Significant correlations between ESTIMATE immune scores and key immune-related marker molecules. **(D)** Boxplots illustrating the ESTIMATE immune score across different immune clusters. Kruskal–Wallis test. **(E)** Boxplots illustrating TMB across different immune clusters. Kruskal–Wallis test. **(F)** Spearman correlation analysis of the TMB and CD4^+^ T-cell infiltration. **(G)** Boxplots illustrating CD4^+^ T-cell infiltration across different immune clusters. **(H)** Survival Kaplan–Meier curves according to immune subtypes. Log-rank test. **(I)** Boxplots illustrating the TIDE score across immune subtypes. **(J)** Volcano plot showing the correlations between different peak events and immune scores. **(K)** Volcano plot showing the *cis*-effect genes at 12p13.31. Spearman’s correlation coefficients, * *p* < 0.05. **(L)** Heatmap showing the immune score, copy number alterations, RNA abundance, and protein abundance of LPCAT3, TPI1 and PHB2. **(M)** Boxplots illustrating the protein abundance of LPCAT3 across different immune clusters. **(N)** Boxplots illustrating the protein abundance of TPI1 across different immune clusters. **(O)** Boxplots illustrating the protein abundance of PHB2 across different immune clusters. The data underlying the graphs shown in the figure can be found in S1 Data.

Immune infiltration analysis demonstrated a strong association between high immune scores and elevated expression of immune regulatory factors, particularly checkpoint molecules (Figs 6C and S7C). Compared with the immune-warm and immune-cold phenotypes, immune-hot tumors exhibited significantly higher ESTIMATE immune scores and enhanced infiltration of effector memory CD4^+^ T cells and macrophages (Fig 6D, 6F, and 6G). Tumor mutational burden and immune infiltration also differed across these three immune phenotypes (Fig 6E). Importantly, patients with immune-hot tumors showed more favorable overall survival than those with immune-warm or immune-cold tumors (Fig 6H). Tumor Immune Dysfunction and Exclusion (TIDE) analysis further showed lower immune evasion scores in the immune-hot phenotype, suggesting that these tumors may be more immunologically active and potentially more responsive to immune checkpoint blockade (Fig 6I). These findings support the biological and clinical relevance of the three immune phenotypes, although further validation in independent cohorts is required [16].

Genomic alterations in immune-related genes within tumors may increase sensitivity to ICIs, facilitating immune evasion. In cervical cancer, CNVs at the 12p13.31 locus were significantly correlated with immune infiltration scores (Fig 6J), supporting prior findings in advanced disease [17]. Cis-acting genomic analysis at this locus identified significant regulatory effects on five downstream proteins, including LPCAT3, TPI1, and PHB2, at the proteomic level (Fig 6K). Positive associations were observed among immune scores, 12p13.31 CNVs, and the RNA and protein levels of these *cis-*effect proteins (Fig 6L). Higher expression and abundance of LPCAT3, TPI1, and PHB2 were specifically enriched in the immune-hot tumor subtype (Figs 6M–6O and S7F–S7H). Collectively, this work deciphers immune complexity among CC subtypes and highlights the link between a key genomic locus and the immunosuppressive tumor microenvironment.

## Discussion

This study presents a comprehensive proteogenomic characterization of cervical cancer (CC) in a Chinese cohort, integrating multi-omics data from tumors and matched NATs. We identified major mutational processes dominated by APOBEC-associated, age/background, and DNA repair-associated signatures. Multi-omics analyses revealed key oncogenic drivers (e.g., PIK3CA, EP300, MPRIP) and pathway activation, with LAMC2 emerging as a promising diagnostic and therapeutic target. Immune profiling delineated three major tumor microenvironment phenotypes (immune-hot, immune-warm, and immune-cold) with distinct immune infiltration, checkpoint expression, TIDE scores, and survival patterns. Our findings elucidate genomic, transcriptomic, and proteomic disruptions and their regulatory relationships, underscoring the value of proteogenomic integration in uncovering novel cancer biology and therapeutic hypotheses for CN-CC patients.

Comparison with previous predominantly North American-European studies indicates that the major molecular features of cervical cancer are largely conserved in our Chinese cohort. The overall tumor mutational burden and the frequencies of recurrent driver alterations, including PIK3CA and EP300, were comparable to those reported by TCGA, and APOBEC-associated mutagenesis together with dysregulation of PI3K/MAPK, cell-cycle, EMT, and immune-related programs was also broadly consistent with previous studies. These findings support substantial cross-population conservation of the core molecular architecture of cervical cancer. At the same time, the present cohort provides additional resolution through paired adjacent non-tumor tissues and deep proteomic and phosphoproteomic profiling. In particular, we identified HPV integration status-associated proteogenomic programs, coordinated CDK-related cell-cycle activity, LAMC2- and IDO1-associated molecular features, and links between 12p13.31 copy-number alterations and the immune microenvironment. These findings should not yet be interpreted as Chinese-specific alterations, because cohort composition, HPV characteristics, histological distribution, and technical platforms may contribute to inter-study differences. Rather, they represent molecular features that were more deeply resolved in this Chinese cohort and warrant validation in ethnically diverse populations using harmonized multi-omics approaches.

Our study, situating its findings within the established context of key somatic mutations and copy number alterations in cervical carcinogenesis [2,18–20], delineates a distinct landscape of somatic CNVs stratified by HPV integration status. We demonstrate that non-integrated cervical carcinoma samples exhibit the highest genomic stability, whereas productive HPV-integrated samples display extensive CNVs, indicative of significant genomic instability [21]. We observed recurrent CNVs in pivotal oncogenes such as PTEN, CD274, and YAP1, corroborating that HPV integration actively sculpts the host genome. The functional consequence is evidenced by the enrichment of DEGs within these CNV regions in cancer-related pathways (Fig 2C). These widespread CNVs appear to drive oncogenic phenotypes through multi-omic dysregulation of key genes. For instance, we identified PFKFB4, whose amplification-driven phosphorylation of SRC-3 promotes proliferation and represents a tangible therapeutic vulnerability [22]. Moreover, analogous studies linking gene loss (e.g., *CD177*) to poor prognosis in other cancers suggest a broader mechanistic impact of CNVs [23,24]. Consequently, pervasive CNVs likely orchestrate the aberrant expression of marker genes, fundamentally influencing tumor initiation, progression, therapy resistance (targeting YAP1-related pathways) [25], and overall patient outcomes.

Multi-omics analysis offers a powerful approach to dissect the functional consequences of CNVs in cervical cancer, revealing how genomic alterations propagate through transcriptional and post-translational regulation. We demonstrate that CNVs modulate gene expression in cis and trans, influencing mRNA, protein, and phosphoprotein levels. For instance, genomic alterations in key drivers such as ACTL6A—known to promote tumor progression by inhibiting ferroptosis in gastric cancer via GCLC upregulation [26]—and SEC62, a recognized prognostic marker in multiple cancers [27], underscore their potential roles in cervical carcinogenesis. Notably, TP63 alterations significantly affected both protein and phosphoprotein abundance, indicating substantial post-translational regulation (Fig 2H and 2I). These findings highlight candidate marker genes and dysregulated pathways, advancing our understanding of cervical tumor biology and offering potential targets for therapeutic intervention and prognostic prediction.

Integrated multi-omics analysis provides a powerful framework for delineating molecular distinctions between cervical tumors and adjacent non-tumor tissues, as well as molecular alterations associated with HPV integration status. HPV integration status was used as the primary biological grouping variable, revealing coordinated transcriptomic, proteomic, and phosphoproteomic differences among non-integrated, silent, and productive tumors. Our study identified several key drivers: SERPINB3, implicated in apoptosis and tumor invasion, showed elevated protein and phosphoprotein levels in tumors and, together with S100A8, correlates with advanced disease and poor prognosis in cervical squamous cell carcinoma [28]. PDCD4, a tumor suppressor targeted by oncogenic microRNA-150, inhibits proliferation and invasion [29]. FHL2 promotes proliferation by enhancing MDM2-mediated degradation of IER3 [30]. Additionally, TFRC, involved in E2F signaling, and PLOD2, which facilitates EMT and metastasis under hypoxia and TGF-β1 induction, were both highly expressed in cervical tumors [31,32]. However, their associations with overall survival were modest and time-dependent. In particular, PLOD2 expression did not clearly distinguish long-term survival after approximately 3,000 days, and TFRC expression did not distinguish early survival risk before approximately 2,000 days. Thus, these survival results should be interpreted cautiously and do not establish TFRC or PLOD2 as robust clinical prognostic biomarkers. Rather, they support the potential biological relevance of these molecules and warrant further validation in larger independent cohorts with multivariable and time-dependent survival analyses. These findings indicate that HPV integration status and viral transcriptional activity are associated with distinct proteogenomic programs, including EMT, hypoxia, interferon response, complement activation, E2F targets, MYC targets, and DNA repair pathways. These HPV-associated molecular alterations may provide mechanistic insights into cervical cancer progression and nominate candidate pathways for future therapeutic investigation.

Elevated expression of LAMC2 is established as a promoter of tumor invasion, metastasis, angiogenesis, and reduced apoptosis [33,34]. Furthermore, genes overexpressed in the high-LAMC2 subgroup of cervical cancer were significantly enriched in key oncogenic pathways, including focal adhesion, PI3K-AKT, HPV infection, and ECM-receptor interaction (Fig 5I). Concurrently, IDO1 contributes to immune escape in cervical squamous cell carcinoma by mediating immune tolerance and suppressing autoimmunity [35]. These collective findings nominate LAMC2 as a candidate tissue-associated marker of extracellular matrix remodeling and invasive tumor biology. However, the present data do not establish LAMC2 as a clinically validated diagnostic, prognostic, or circulating biomarker. Further validation in independent cohorts, preferably using standardized tissue-based assays such as immunohistochemistry, will be required to determine whether LAMC2 can support pathological risk stratification or therapeutic development.

Our analysis identifies several transcription factors with altered activity in cervical cancer. Activities of E2F1, E2F4, MYC, and MNT are significantly activated, while POU5F1, PBX3, and KLF9 are downregulated (Fig 3D and 3E). Among these, the E2F family, centrally regulated through the CDK-RB-E2F axis [36], is critical for cell cycle control. Notably, E2F1 is frequently overexpressed in tumors; its dysregulation disrupts downstream pathways, promoting tumor initiation and progression [37]. The collective activation of these TFs underpins key oncogenic processes in cervical cancer.

Dysregulation of protein kinases plays a pivotal role in oncogenesis by driving uncontrolled proliferation, invasion, and metastasis [11]. Among them, CDK2 is frequently activated across human cancers and is considered a critical regulator of tumorigenesis [38]. Similarly, CDK7 has emerged as a key promoter of tumor progression in multiple cancer types and represents a promising therapeutic target [39,40]. These findings underscore the importance of investigating CDK family members in cervical cancer to advance targeted treatment strategies.

HPV-associated cervical cancer develops within a complex antigenic and inflammatory tumor microenvironment. In our cohort, immune-hot tumors showed increased expression of checkpoint-related molecules, including PDCD1, CD274, CTLA4, IDO1, and LAG3, together with cytotoxic markers such as GZMA, GZMB, and PRF1. PDCD1 expression likely reflects activated and/or exhausted tumor-infiltrating T cells, whereas CD274 expression may originate from malignant epithelial cells as well as non-malignant components of the tumor microenvironment, including myeloid-lineage cells and antigen-presenting cells. Therefore, we interpret the co-elevation of PDCD1 and CD274 as a feature of an immune-inflamed and checkpoint-regulated microenvironment, rather than as direct evidence that persistent HPV infection alone induces PD-1/PD-L1 expression (S7I Fig) [28,35]. Consistent with this phenotype, our analysis demonstrated that the immune-hot subgroup exhibits a significantly more favorable prognosis (Fig 6H) and lower TIDE scores, suggesting a potentially better response to ICIs (Fig 6I). Furthermore, we identified that copy number alterations in specific genomic loci, such as 12p13.31, correlate with immune scores (Fig 6J). These findings underscore the critical link between genomic instability and immune regulation. Mechanistically, driver genes including LPCAT3, which modulates lipid metabolism and ferroptosis, and TPI1, an activator of the oncogenic PI3K-AKT-mTOR pathway, may critically shape the immune landscape [41–45]. In summary, a comprehensive analysis of immune cell infiltration and associated genomic alterations is essential for stratifying patients, predicting immunotherapy response, and advancing personalized therapeutic strategies in cervical cancer.

In summary, this study delineates a comprehensive proteogenomic landscape of cervical cancer in the Chinese population through integrated analysis of genome, transcriptome, proteome, and phosphoproteome data. It identifies dominant pathway alterations that correlate with distinct proteomic subtypes, revealing potential molecular mechanisms underlying clinical phenotypes and patient outcomes. Our findings support substantial cross-population conservation of the major genomic drivers of cervical cancer while extending previous studies by providing deeper proteomic and phosphoproteomic resolution in an Asian cohort. While clinical validation of these derived therapeutic hypotheses remains for future study, the findings establish a foundational framework for advancing molecularly guided precision medicine in cervical cancer. Collectively, this work not only characterizes the molecular drivers of disease phenotypes but also provides a substantial bioinformatics resource to support future translational and hypothesis-driven research.

## Materials and methods

### Ethics statement

This study was reviewed and approved by the Institutional Review Board of Tongji Hospital, Tongji Medical College, Huazhong University of Science and Technology (TJ-IRB20210535). Informed consent was obtained from all patients, and sample collection was performed following standard diagnostic procedures without imposing any additional burden on the patients. Clinical data for the cohort is provided in S1 Table.

### Specimen preparation

For sample quality control of tumor samples, the following selection criteria were applied: (1) For tumor cellularity assessment, three frozen sections (top, middle, and bottom) from each tissue block were resected and subjected to H&E staining. All H&E-stained slides were scanned and analyzed pathologically; (2) Only samples with at least 60% tumor cell nuclei and less than 10% necrosis were retained; (3) Histological evaluation of all tumor samples was independently performed by two experienced pathologists.

For quality control of the non-tumor tissue (NAT) samples, the following criteria were used for sample selection: (1) Collected from regions at least 3 cm away from the tumor margin during surgical resection; (2) Tumor cellularity analysis, three frozen sections (top, middle, and bottom) of each tissue block were resected and subjected to H & E staining. All H & E-stained slides were scanned and submitted for pathological analysis; (3) Histopathological examination confirmed the absence of tumor infiltration and high-grade intraepithelial lesions (HSIL/CIN2+); (4) The histological assessment of all tumor samples was accomplished by two experienced pathologists separately.

### HPV typing and classification of HPV integration status

HPV genotype information and HPV integration/transcriptional activity annotations were obtained from our previously published study, Multi-omics characterization of silent and productive HPV integration in cervical cancer [46]. Briefly, for VCS data, cleaned reads were aligned to the human-HPV reference using BWA-MEM and deduplicated with Picard. HPV genotypes were identified using a mean viral genome read depth ≥10, and SurVirus-detected integration breakpoints supported by ≥10 reads were retained. For bulk RNA-seq, reads were aligned using STAR, HPV abundance was quantified with Subread, and HPV types with CPM ≥1 were retained. Virus-host fusion transcripts were identified from STAR chimeric alignments, requiring ≥10 supporting reads. HPV typing and HPV integration analyses were performed using HPV-aware sequencing analyses with HPV reference genomes obtained from public databases. DNA-level HPV integration was determined based on virus-host chimeric evidence, whereas RNA-level transcriptional activity was assessed by detecting HPV transcripts and virus-host fusion transcripts from RNA-seq data. Tumors with DNA-level HPV integration and detectable HPV transcriptional activity were classified as having productive HPV integration; tumors with DNA-level HPV integration but without detectable HPV transcriptional activity were classified as having silent HPV integration; and tumors without evidence of HPV integration were classified as non-integrated. In the present study, WES data were used for human genomic alteration analysis and were not used alone to infer HPV integration status.

### Sample processing for DNA and RNA extraction

DNA and RNA were extracted from tumor and adjacent normal samples via QIAGEN’s AllPrep DNA/RNA Mini Kit. DNA concentrations were measured with a Qubit DNA Assay Kit with a Qubit 2.0 fluorimeter. Samples that yielded sufficient DNA and passed quality control were sequenced. RNA concentrations were measured using a Qubit, and quality was assessed with an Agilent 2100. Only RNA samples that passed quality control with a minimum RNA integrity number (RIN) score of 7 were submitted for sequencing.

### Whole-exome sequencing

Library construction was carried out using 1 µg of DNA per sample with the Agilent SureSelect Human All Exon V6 Kit (Agilent Technologies, CA, USA) according to the manufacturer’s protocol. In brief, DNA was fragmented to 180–280 bp using a Covaris hydrodynamic shearing system (Massachusetts, USA), followed by end repair, A-tailing, adaptor ligation, and library enrichment PCR with Kapa Hyper-Prep reagents. The libraries were hybridized with biotin-labeled probes in liquid phase, captured using streptavidin-coated magnetic beads to isolate gene exons, and enriched via PCR to add index tags for sequencing. Products were purified with the AMPure XP system (Beckman Coulter, Beverly, USA) and quantified using the Agilent High Sensitivity DNA Assay on the Agilent Bioanalyzer 2100 system.

Clustering of the index-coded samples was conducted following the manufacturer’s protocol. After cluster generation, the DNA libraries were sequenced on the Illumina NovaSeq platform, producing 150 bp paired-end reads.

### Bulk RNA sequencing

Library construction was performed using 2 µg of RNA per sample with the VAHTS mRNA-seq v2 Library Prep Kit for Illumina, following the manufacturer’s protocol. Briefly, mRNA was purified from total RNA using poly-T oligo-attached magnetic beads, followed by fragmentation with fragmentation buffer. First- and second-strand cDNA synthesis was carried out, and the resulting overhangs were converted into blunt ends. After adenylation of the 3′ends, adaptors with hairpin loop structures were ligated, followed by PCR amplification. The final library, approximately 350 bp in size, was quality-checked using the Qubit HS system and an Agilent 2100 Bioanalyzer/Fragment Analyzer 5300. The libraries were sequenced on the Illumina NovaSeq platform to produce 150 bp paired-end reads, following the manufacturer’s guidelines.

### Protein extraction

For each protein extraction, approximately 3 mg of wet tissue was used, which yielded sufficient protein for downstream enzymatic digestion and LC–MS/MS analysis. The sample was grinded by liquid nitrogen into cell powder and then transferred to a 5-mL centrifuge tube. Four volumes of lysis buffer (8 M urea, 1% protease inhibitor cocktail, 1% phosphatase inhibitor cocktail) were added, followed by sonication on ice three times using a high-intensity ultrasonic processor (Scientz). The debris was removed by centrifugation at 12,000*g* for 10 min at 4 °C. The supernatant was then collected, and the protein concentration was measured using a BCA kit, following the manufacturer’s instructions.

For protein digestion, the sample was precipitated with TCA at 4 °C for 2 hours, then washed 2–3 times with acetone and resuspended in 200 mM TEAB. Trypsin was added at a 1:50 enzyme-to-protein ratio for an overnight digestion. The protein mixture was reduced to 10 mM DTT for 1 hour at 37 °C and alkylated with 20 mM IAA for 45 min at room temperature in the dark. A second digestion was performed by adding trypsin at a 1:100 ratio for an additional 4 hours.

### TMT labeling

Following trypsin digestion, the peptides were desalted using a Strata X C18 SPE column (Phenomenex) and vacuum-dried. The peptides were reconstituted in 0.5 M TEAB and processed according to the 11-PLEX TMT kit protocol. Briefly, one unit of TMT reagent, sufficient to label 100 µg of protein, was thawed and reconstituted in 24 µL of buffer. The peptide mixtures were incubated at room temperature for 2 hours, pooled, desalted, and vacuum-dried. A mixed reference sample was included in each 11-plex to enable quantitative comparisons across experiments.

### HPLC fractionation

To reduce sample complexity, the peptide samples were fractionated using high-pH reverse-phase HPLC on an Agilent 300Extend C18 column (5 µm particles, 4.6 mm ID, 250 mm length). A total of 3.3 mg of peptides were separated over 80 min with a 2%–60% acetonitrile gradient in 10 mM ammonium bicarbonate (pH 10), resulting in 80 fractions. These fractions were then combined into 12, with 10% of each fraction reserved for proteome analysis, while the remaining 90% were further combined into 6 fractions for phosphopeptide enrichment.

### Affinity enrichment

The peptide mixtures were initially incubated with IMAC microspheres in a loading buffer (50% acetonitrile/6% trifluoroacetic acid) under agitation. After centrifugation, the phosphopeptide-enriched IMAC microspheres were collected, and the supernatant was discarded. The IMAC microspheres were washed sequentially with 50% acetonitrile/6% trifluoroacetic acid and 30% acetonitrile/0.1% trifluoroacetic acid to remove nonspecifically bound peptides. Phosphopeptides were then eluted from the IMAC microspheres using an elution buffer containing 10% NH_4_OH, followed by lyophilization of the supernatant for LC–MS/MS analysis.

### LC–MS/MS analysis

Peptides were analyzed using a nanoelectrospray ionization (NSI) source followed by tandem mass spectrometry (MS/MS) on a Q Exactive HF-X (Thermo) system, coupled with an EASY-nLC 1200 UPLC. For peptide separation, the gradient was increased from 6% to 22% solvent B (0.1% formic acid in 90% acetonitrile) over 38 min, from 22% to 32% over 14 min, then ramped to 80% in 4 min, and held at 80% for 4 min. For phosphoproteome analysis, the gradient was increased from 3% to 20% solvent B over 88 min, from 20% to 32% over 24 min, ramped to 80% in 4 min, and held at 80% for 4 min.

An electrospray voltage of 2.2 kV was applied, and intact peptides were detected in the Orbitrap at a resolution of 120,000. Peptides were selected for MS/MS with a normalized collision energy (NCE) of 28, and fragments were detected at a resolution of 45,000. A data-dependent acquisition alternated between one MS scan and 15 MS/MS scans, with a dynamic exclusion of 30.0s. The automatic gain control (AGC) target was set to 5 × 10^4^, with an intensity threshold of 5,000 and a maximum injection time of 90 ms. The first fixed mass was set at 100 *m*/*z*.

### Proteomics database search analysis

The MaxQuant search engine was employed for the standard database search of all MS/MS raw data [47]. MS/MS spectra were matched against the human Swiss-Prot database, containing 20,431 sequences (downloaded in August 2019) along with 475 Swiss-Prot HBV protein sequences. Trypsin/P was specified as the cleavage enzyme, allowing up to 2 missed cleavages. The precursor ion mass tolerance was set to 20 ppm for the first search and 4.5 ppm for the main search, while the fragment ion mass tolerance was 0.02 Da. The minimum peptide length was set to 7 amino acids. Fixed modification was set as carbamidomethylation on cysteine, with oxidation on methionine, acetylation at the protein N-terminus, and deamidation (NQ) as variable modifications. Phospho (STY) was specified for phosphoproteome searches. The false discovery rates (FDR) for peptide-spectrum matches (PSMs) and proteins were set to <1%, and the minimum score for modified peptides was >40. The TMT 11-plex method was selected for quantification, and all other MaxQuant parameters were left at default settings.

### Data normalization

The TMT-based intensity matrix of proteins or phosphorylation sites was extracted from the MaxQuant output files for all batches. For each protein or phosphorylation site, the TMT ratio was calculated by dividing the intensity by that of the internal reference cell line from the same batch. Proteomic and phosphoproteomic data for each sample were then normalized using the median centering method. After normalization, the normalized abundance values (NAVs) of proteins or phosphorylation sites followed a log2 normal distribution centered around zero.

### Read alignment

After the exclusion of low-quality reads, the WES reads were aligned to GRCh38 with BWA (v0.7.17) [48]. Defaults were used in bwa mem, with the exception that for bwa mem, 10 threads were utilized (-t 10).

### Read duplication marking

Reads duplicates were marked per library via Picard MarkDuplicates (v2.23.8). Finally, each per-library BAM with duplicates marked was merged together to generate a single BAM file for the sample. All other parameters were set to defaults.

### Somatic variant calling for paired and tumor-only WES samples

WES data were available for 98 tumor samples. Among them, 81 tumors had matched adjacent normal tissue WES data and were analyzed using a paired tumor-normal somatic variant-calling strategy. The remaining 17 tumors did not have matched normal WES data and were analyzed using a tumor-only strategy. For these tumor-only samples, we constructed a cohort-specific normal baseline from all available normal WES samples generated in this study using the same sequencing platform, capture kit, and bioinformatic pipeline. Recurrent variants detected in normal samples were used to define a normal baseline and were excluded from tumor-only variant calls. Candidate variants were further filtered according to sequencing depth, variant allele fraction (VAF), mapping quality, strand bias, read-position bias, and population allele frequency in public germline databases, where applicable.

Variants retained after these filtering steps were considered putative somatic variants for the 17 tumor-only samples. Because matched germline DNA was unavailable for these cases, we acknowledge that residual private germline variants cannot be completely excluded. Therefore, tumor-only variant calls were interpreted with caution and were subjected to more stringent filtering than paired tumor–normal calls.

### Somatic mutation calling

After the exclusion of low-quality reads, WES reads were mapped to the GRCh38 reference sequence with BWA (v0.7.17) [48]. PCR duplicates were removed via Picard (v2.23.8). Somatic variants were detected via Strelka (v2) [49], VarScan2 (v2.4.4) [50], SomaticSniper (v1.0.5.0) [51], and GATK (v4.4.2.0) [52] on exome data of tumors and matched adjacent pairs. Annotation of variants was performed via ANNOVAR (version 2017) [53]. Germline variants were filtered from the 1000 Genomes database, the NHLBI Exome Sequencing Project (ESP6500) and the Exome Aggregation Consortium (EXAC). A stringent downstream filter comprising the following criteria was used to obtain high-quality somatic variants: a minimum of 8× coverage, a VAF ≥5%, at least 5 variant-supporting reads in the tumor sample, and a VAF < 2% in the matched adjacent sample.

To accurately identify somatic mutations in tumor samples, we used four methods: Strelka (v2), VarScan2 (v2.4.4), SomaticSniper (v1.0.5.0), and GATK (v4.4.2.0). We retained single nucleotide variants (SNVs) identified by any two of these four methods and insertion/deletion mutations (indels) identified by any two of the three methods: Strelka (v2), VarScan2 (v2.4.4), and GATK (v4.4.2.0). For the merged SNVs and indels, we applied an 8× coverage cutoff for tumors and normal tissues at the same time. We also filtered SNVs and indels by a minimal variant allele frequency (VAF) of 0.05 in tumors and a maximal VAF of 0.02 in normal samples. Finally, we filtered any SNV that was within 10 bp of an indel found in the same tumor sample.

### Somatic copy number alteration (SCNA) analysis

For each tumor, SCNAs were inferred via CNVkit (v0.9.7) with default parameters [54]. Segment-level ratios were calculated and log2-transformed. Significant focal SCNAs across all samples were identified via Genomic Identification of Significant Targets in Cancer (GISTIC v2.0) to determine which SCNA regions were significantly gained or lost by chance, with a *q* value ≤0.1. A log2 ratio cutoff of ±0.9 was used to define SCNA amplification and deletion [55].

### Significantly mutated gene analysis

All somatic variants were first annotated via ANNOVAR (version 2017). To identify SMGs in the CN-CC cohort, the filtered mutations were further used to identify SMGs via MutSigCV (version 1.4.1) with the default parameters [56]. The final MutSigCV *p* values were converted to *q* values via the method of Benjamini and Hochberg, and genes with *q* values ≤0.1 were considered significantly mutated.

### Mutation signature analysis

To uncover mutational processes active in CN-CC patients, the de novo mutational signatures of all 98 tumors were determined via the SomaticSignatures (v.2.32.0) R package. The number of signatures was determined by choosing the reflection point in the curves of explained variance and residual sum of squares (RSS). The 96 mutational vectors (or contexts) generated by somatic SNVs on the basis of six base substitutions (C > A, C > G, C > T, T > A, T > C, and T > G) within the 16 possible combinations of neighboring bases for each substitution were used as input data to infer their contributions to observed mutations. Proportions were used to cluster samples via hierarchical clustering with “correlation” distance and an average linkage function via the pvclust (v.2.2-0) R package [57].

### HRD score estimation

Allele-specific copy-number profiles were inferred from paired tumor-normal WES BAM files using Sequenza and then used as input for scarHRD [58]. HRD scores were calculated using the scarHRD package. Briefly, allele-specific copy-number profiles were first generated from paired tumor-normal WES data and then used as input for scarHRD. scarHRD estimates three genomic scar components: loss of heterozygosity (HRD-LOH), telomeric allelic imbalance (TAI), and large-scale state transitions (LST). The final HRD score was defined as the sum of the LOH, TAI, and LST scores. Samples lacking matched normal WES data were excluded from HRD score estimation because tumor-only sequencing does not provide a reliable germline baseline for distinguishing somatic allelic imbalance from inherited copy-number variation or mapping artifacts. Therefore, HRD scores were calculated for 81 paired tumor-normal WES samples, whereas the 17 tumor-only WES samples were not assigned HRD scores. Samples with HRD scores >30 were considered to have moderate-to-high HRD in this analysis.

### CNV-driven *cis-* and *trans-*effects

CNVs affecting protein and phosphoprotein abundance in either “*cis*” (within the same aberrant locus) or “*trans*” (remote locus) mode were visualized via the “multiOmicsViz” R package. Spearman correlation coefficients and associated multiple-test adjusted *p* were calculated for all CNV-protein and CNV-phosphoprotein pairs.

### RNA-seq read mapping and normalization

The quality of the raw RNA-seq data from 79 CC and paired NATs was assessed, and the adaptors were trimmed via fastp (v 0.21.0) [59]. The raw RNA-seq reads were aligned to the human reference genome (GRCh38.p13 assembly) via HISAT2 (v2.2.1) [60]. The mapped reads were assembled into transcripts or genes via StringTie (v2.2.1) [60], and a genome annotation file (gencode.v35.annotation.gtf). For mRNA expression quantification, the relative abundance of transcripts was then normalized via the fragments per kilobase of transcript per million mapped reads (FPKM) method.

### Transcription factor activity inference

TF activity was inferred via the Gene set enrichment analysis (GSEA) algorithm on log2-transformed RNA data [61]. The TF targets were collected from DoRothEA, and the medium confidence targets (A, B, C) were used for further analysis. The relative activity scores for CCs and NATs were represented with NES, and the *p* value was adjusted via the FDR method. TFs with an FDR < 0.05 were considered significant.

### Proteomic and phosphoproteomic subtyping via NMF

For proteomic and phosphoproteomic subtyping, the top 3,000 most variable coding proteins were chosen from the expression protein and phosphoproteomic matrix among the tumor samples in our CN-CC cohorts. To identify robust clusters, unsupervised clustering of nonnegative matrix factorization was implemented in the R environment. The optimal rank parameters were first determined via 50 iterations ranging from 2 to 10 (*K* = 2, 3, 4, 5, 6, 7, 8 and 9) with default settings. On the basis of the plot, we selected a 3-cluster as the best solution for the consensus matrix, with *K* = 3 deemed to be the cleanest separation among the clusters. We then perform 50 iterations with the optimal ranks (*K* = 3) to acquire the final NMF clustering solutions.

### Gene set enrichment analysis (GSEA)

GSEA pathway enrichment analysis was performed via the clusterProfiler R package (v4.4.4) [62], and the functionally annotated pathways were implemented in ggplot2 (v3.4.3). The gene sets “h.all.v7.5.1.symbols.gmt” and “c2.all.v7.5.1.symbols.gmt” used for GSEA in this study were downloaded from the MSigDB database [63].

### Identification of immune clusters

The abundances of 64 different cell types in 112 CN-CCs were computed via xCell [15]. For this analysis, the gene expression matrix, excluding >50% missing values across all the samples, was utilized. S1 Table includes the final xCell scores for different cell types for the 112 CN-CCs. Consensus clustering was performed on the basis that cells were detected in at least 30% of the patients (adjusted *p* < 0.01). This filtering resulted in 40 cell types. To identify sample groups with similar immune/stromal characteristics, consensus clustering was performed via the R package ConsensusClusterPlus on the basis of the normalized Z scores of the 40 xCell signatures selected above [64]. Specifically, 80% of the original 112 samples were randomly subsampled without replacement and partitioned into six major clusters via the partitioning around medoids (PAM) algorithm.

### Estimation of stromal and immune scores

ESTIMATE was also used to infer tumor purity and immune and stromal scores on the basis of RNA-seq and global proteomic data, with default algorithm parameters [65].

### Analysis of immune-related pathways

To investigate the impact of enrichment of different biological process pathways on immune clusters, the “GSVA” R package was used to conduct GSVA enrichment analysis [66]. For this analysis, the gene sets “h.all.v7.5.1.symbols.gmt” and “c2.all.v7.5.1.symbols.gmt” for GSVA analysis were downloaded from the MSigDB database. Pathway scores of 112 tumors were computed on the basis of proteomic data with transformed Z scores.

### Statistical analysis

All data analyses were conducted in R v4.2.1. The Wilcoxon rank-sum test was utilized to compare variables across multiple groups, with a significance level of 0.05 assumed.

## Supporting information

S1 Fig Mutation profiles of the CN-CC cohort, related to Fig 1. **(A)** Representative hematoxylin and eosin (H&E) staining images of cervical cancer tissues and matched normal adjacent tissues (NATs) used for multi-omics profiling. Scale bars, 100 μm. **(B)** Distribution of mutation burdens in each cancer type. Each dot corresponds to the mutation burden calculated from a tumor-normal pair. **(C)** Lollipop plot visualizing all protein-altering somatic mutations detected in this cohort. The size of a lollipop represents the number of samples with corresponding mutations, and the color represents a specific type of mutation. EP300 gene. **(D)** Four clusters identified by NMF. **(E)** Upper, residual sum of squares (RSS) of different signature number selections. The percentage of variance explained by different signature number selections is lower. **(F)** Thirteen mutation signatures were detected in the CN-CCs. **(G)** Unsupervised clustering results between the COSMIC signatures and 13 signatures of the CN-CC cohort. **(H)** Constrained refitting of reconstructed sample spectra. **(I, J)** Contributions of 13 mutation signatures in different stages and sample types. * *p* < 0.05. The data underlying the graphs shown in the figure can be found in S2 Data.
(TIF)

S2 Fig Proteogenomic consequences of genetic aberrations, related to Fig 2. **(A)** Genome-wide focal amplification. **(B)** Heatmap of the CNAs of 98 CN-CC tumor samples: red and blue represent copy gain and loss, respectively, in units of log2 (tumor/NAT). The *x* axis represents the 98 tumor samples. **(C)** Genome-wide focal deletions. Chromosomal locations of peaks of significantly recurring focal amplifications (red) and deletions (blue) were filtered by FDRs. **(D)** A list of cancer-related genes that were differentially expressed between the CNV and WT groups (Wilcoxon rank-sum test, * *p* < 0.05, ** *p* < 0.01, *** *p* < 0.001, **** *p* < 0.0001). (**E–G**) Correlations of CNV (x axes) with mRNA, protein and phosphoprotein abundance levels (y axes) highlight CNV *cis*- and *trans*-effects. Significant (adjusted *p* < 0.05, Spearman’s correlation) positive (red) and negative (blue) correlations between CNV and mRNAs, proteins or phosphoproteins are indicated. **(H)** Genes with CNV *cis*-effects (adjusted *p* < 0.05, Spearman’s correlation) at the mRNA, protein, and phosphoprotein levels. **(I)** Enrichment analysis of overlapping genes of *cis* CNV-mRNAs and CNV-proteins correlations (adjusted *p* < 0.05, Spearman’s correlation). The data underlying the graphs shown in the figure can be found in S2 Data.
(TIF)

S3 Fig Integrated multiomics analyses of tumor tissues compared with NATs, related to Fig 3. **(A–C)** PCA plots of the transcriptomic, proteomic and phosphoproteomic abundances in tumors (red) and NATs (green). **(D)** Gene-wide correlations of mRNA and protein expression in tumors (red) and NATs (green). **(E)** Differentially expressed transcriptome profiles in tumor and NAT samples. **(F)** Differentially expressed phosphoprotein profiles in tumor and NAT samples. **(H, J)** Enrichment analysis of the list of significantly differentially expressed genes (adjusted *p* < 0.05) between tumors and NATs. **(G, I, K)** Cluster diagram representing pathways significantly associated with upregulated genes in tumor samples. The Metascape output represents enriched biological concepts as nodes, aggregates those nodes into clusters on the basis of the similarity of their protein membership, and names the clusters on the basis of their most significant node. The node size represents the number of differentially expressed gene products. The terms with the best *P* values from each of the 20 clusters. **(L)** Box diagram showing the mRNA expression of four transcription factors (E2F1, E2F4, KLF9 and PBX3) in tumor and NAT samples. The data underlying the graphs shown in the figure can be found in S2 Data.
(TIF)

S4 Fig Relationships between protein kinase and substrate protein phosphorylation, related to Fig 3. **(A)** Volcano plot showing the downstream phosphosites for kinase activity. **(B)** Spearman correlation between kinase substrate phosphorylation site abundance and kinase protein abundance. **(C)** Spearman correlation between kinase protein abundance and pathway score. The data underlying the graphs shown in the figure can be found in S2 Data.
(TIF)

S5 Fig Comparative proteogenomic characteristics of cervical cancer subgroups, related to Fig 4. **(A–C)** Consensus matrices of identified clusters (*K* = 3). The data underlying the graphs shown in the figure can be found in S2 Data.
(TIF)

S6 Fig Identification of early proteomic detection and therapeutic biomarkers, related to Fig 5. **(A, B)** Relative protein abundance and mRNA expression of STAT1 and PARP1 between tumors and NATs (Wilcoxon rank-sum test) as well as across cervical cancer subgroups (Kruskal–Wallis test). **(C)** Relative protein abundance and mRNA expression of MKI67 between tumors and NATs (Wilcoxon rank-sum test) as well as across cervical cancer subgroups (Kruskal–Wallis test). The line and box represent the median and upper and lower quartiles, respectively. **(D)** Spearman correlation between the mRNA expression and protein abundance of LAMC2 and IDO1. **(E)** LAMC2 and IDO1 expression across three GEO cohorts. **(F, H)** The top 20 genes with significant changes after performing gene perturbation analysis (LAMC2 or IDO1) using scTenifoldKnk on the top 500 highly variable genes. The length of each bar represents the log Fold Change (logFC) of the corresponding gene, and the color gradient from light yellow to dark purple indicates an increasing degree of change. **(G, I)** A ridge plot of GSEA functional enrichment analysis based on genes ranked by the standardized perturbation strength (*Z*-value). Each ridge corresponds to a significantly enriched KEGG pathway. The x-axis represents the gene perturbation strength (*Z*-value), indicating the extent to which genes are affected after virtual knockout of the target gene (LAMC2 or IDO1). The data underlying the graphs shown in the figure can be found in S2 Data.
(TIF)

S7 Fig Immune landscape of the CN-CC cohort, related to Fig 6. **(A)** The heatmap panel shows several key upregulated signaling pathways across different immune clusters. **(B)** Stacked bar plot showing the proportions of distinct HPV infection subtypes distributions in each immune subtype. **(C)** Stacked bar plot showing the proportions of distinct HPV infection subtypes distributions in three immune subtypes. **(D)** Stacked bar plot showing the proportions of proteomic subtype distributions in each immune subtype. **(E)** Boxplots illustrating the ESTIMATE immune score across HPV infection group (Wilcoxon rank-sum test). **(F–H)** Boxplots illustrating the mRNA expression of *LPCAT3*, *TPI1* and *PHB2* across different immune group (Kruskal–Wallis test). **(I)** Correlation of PDCD1/CD274 with immune marker genes. The data underlying the graphs shown in the figure can be found in S2 Data.
(TIF)

S1 Table Clinical information of 112 samples in CCs. (XLSX)

S2 Table The relationship between CN-CC identified mutational signature and COSMIC-curated mutational signatures. (XLSX)

S3 Table Comparison of TF activity in transcriptomic data between tumors and NATs. (XLSX)

S4 Table The downstream phosphosites for CDK2 kinase activity. (XLSX)

S1 Data The individual numerical values in Figs 1C, 1D, 1G, 1H, 2A, 2B, 2D, 2F–2I, 3B–3D, 3G, 3J–3M, 4B–4D, 4F–4H, 5B–5G, 5I, and 6A–6O. (XLSX)

S2 Data The individual numerical values in S1B, S1D–S1J, S2A–S2I, S3A–S3L, S4A–S4C, S5A–S5C, S6A–S6I, and S7A–S7I Figs. (XLSX)

## Abbreviations

AGC: automatic gain control
CC: cervical cancer
CNVs: copy number variations
EMT: epithelial-mesenchymal transition
EXAC: the Exome Aggregation Consortium
FDR: false discovery rates
GSEA: Gene set enrichment analysis
HPV: human papillomavirus
HRD: Homologous recombination deficiency
ICI: immune checkpoint inhibitor
KSEA: Kinase-substrate enrichment analysis
LST: large-scale state transitions
NAT: normal adjacent tissue
NAVs: normalized abundance values
NCE: normalized collision energy
NSI: nanoelectrospray ionization
PAM: partitioning around medoids
PCA: principal component analysis
PLOD2: procollagen-lysine, 2-oxoglutarate 5-dioxygenase 2
PSMs: peptide-spectrum matches
RIN: RNA integrity number
RSS: residual sum of squares
SCNA: Somatic copy number alteration
SMGs: significantly mutated genes
SNVs: single nucleotide variants
TAI: telomeric allelic imbalance
TCGA: The Cancer Genome Atlas
TF: transcription factor
TIDE: Tumor Immune Dysfunction and Exclusion
TMT: tandem mass tag
VAF: variant allele frequency
WES: Whole-exome sequencing
