# Supplementary material for: Integrated multi-omics analysis reveals the molecular landscape and therapeutic strategies in Chinese cervical cancer patients
Source: PLoS Biol. 2026 Oct 5;24(10):e3004029. doi: 10.1371/journal.pbio.3004029 (PMC13637895; doi:10.1371/journal.pbio.3004029)
Supplement: Supplementary file 2 [file pbio.3004029.s017.docx]

***Response to Reviews: 1***

*The authors have done an admirable of correcting the over-interpretations in the original submission, most usually by qualifying potential inferences as requiring further clinical validation in independent cohorts.  They have also clarified that the current emphasis is on understanding biological mechanisms rather than identifying and validating therapeutic targets. They have also added important information about the pathological review of the samples, and the criteria for acceptable cancer and normal adjacent tissues.*

**Reply:** Thank you very much for your positive comments and suggestions, which greatly helped us to improve the manuscript. The manuscript has been carefully revised. We hope that your comments have been addressed accurately.

*The one further revision that would substantially improve the manuscript is to call out explicitly, in the Discussion, how the results in this multi-omic profiling of Chinese patients differed from prior work done in mostly North American - European populations.  From reading the manuscript, it appears most results were concordant between TCGA and the current study, and that is a perfectly acceptable answer that does not detract from the importance of the current work.  However, since a major justification for the current study is the under-study of Asian populations, the Discussion is obligated to point out ways in which the Chinese population resembles other studies, and ways in which the Chinese population appears to be unique.*
**Reply:** We thank you for this important and constructive suggestion. Given the underrepresentation of Asian patients in previous large-scale cervical cancer studies, the Discussion should more explicitly distinguish findings that are conserved across populations from those that appear distinctive in our Chinese cohort.

We have therefore added a dedicated paragraph to the Discussion comparing our findings with those reported in predominantly North American and European cohorts, particularly the studies by Ojesina et al., TCGA, and Chakravarthy et al. Overall, the genomic landscape of our Chinese cohort was highly concordant with previous studies. The frequencies of the major recurrently mutated genes, including PIK3CA and EP300, as well as the overall tumor mutational burden, were comparable to those observed in TCGA. APOBEC-associated mutagenesis and recurrent alterations affecting PI3K/MAPK, cell-cycle, EMT, and immune-related programs were also broadly consistent with previously reported cervical cancer biology.

At the same time, our study extends these observations through substantially deeper proteomic and phosphoproteomic profiling and the availability of matched adjacent non-tumor tissues. These analyses revealed HPV integration status-associated proteogenomic programs, coordinated CDK2/CDK7-related cell-cycle activity, LAMC2- and IDO1-associated molecular features, and genomic–immune associations such as the 12p13.31-related program. We now emphasize that these findings should be regarded as features identified or more deeply resolved in this Chinese cohort, rather than definitive ethnicity-specific alterations, because differences in cohort composition, histological distribution, HPV characteristics, and profiling platforms may also contribute to apparent differences between studies.

We have revised the Discussion accordingly to make clear that a major conclusion of our study is the substantial conservation of core cervical cancer molecular features across populations, while the additional proteomic and phosphoproteomic layers provide complementary insights that were not accessible in earlier predominantly genomic studies.

***Page 11:***

**Discussion**

This study presents a comprehensive proteogenomic characterization of cervical cancer (CC) in a Chinese cohort, integrating multi-omics data from tumors and matched NATs. We identified major mutational processes dominated by APOBEC-associated, age/background, and DNA repair-associated signatures. Multi-omics analyses revealed key oncogenic drivers (e.g., PIK3CA, EP300, MPRIP) and pathway activation, with LAMC2 emerging as a promising diagnostic and therapeutic target. Immune profiling delineated three major tumor microenvironment phenotypes (immune-hot, immune-warm, and immune-cold) with distinct immune infiltration, checkpoint expression, TIDE scores, and survival patterns. Our findings elucidate genomic, transcriptomic, and proteomic disruptions and their regulatory relationships, underscoring the value of proteogenomic integration in uncovering novel cancer biology and therapeutic hypotheses for CN-CC patients.

Comparison with previous predominantly North American-European studies indicates that the major molecular features of cervical cancer are largely conserved in our Chinese cohort. The overall tumor mutational burden and the frequencies of recurrent driver alterations, including PIK3CA and EP300, were comparable to those reported by TCGA, and APOBEC-associated mutagenesis together with dysregulation of PI3K/MAPK, cell-cycle, EMT, and immune-related programs was also broadly consistent with previous studies. These findings support substantial cross-population conservation of the core molecular architecture of cervical cancer. At the same time, the present cohort provides additional resolution through paired adjacent non-tumor tissues and deep proteomic and phosphoproteomic profiling. In particular, we identified HPV integration status-associated proteogenomic programs, coordinated CDK-related cell-cycle activity, LAMC2- and IDO1-associated molecular features, and links between 12p13.31 copy-number alterations and the immune microenvironment. These findings should not yet be interpreted as Chinese-specific alterations, because cohort composition, HPV characteristics, histological distribution, and technical platforms may contribute to inter-study differences. Rather, they represent molecular features that were more deeply resolved in this Chinese cohort and warrant validation in ethnically diverse populations using harmonized multi-omics approaches.

***Page 15:***

In summary, this study delineates a comprehensive proteogenomic landscape of cervical cancer in the Chinese population through integrated analysis of genome, transcriptome, proteome, and phosphoproteome data. It identifies dominant pathway alterations that correlate with distinct proteomic subtypes, revealing potential molecular mechanisms underlying clinical phenotypes and patient outcomes. Our findings support substantial cross-population conservation of the major genomic drivers of cervical cancer while extending previous studies by providing deeper proteomic and phosphoproteomic resolution in an Asian cohort. While clinical validation of these derived therapeutic hypotheses remains for future study, the findings establish a foundational framework for advancing molecularly guided precision medicine in cervical cancer. Collectively, this work not only characterizes the molecular drivers of disease phenotypes but also provides a substantial bioinformatics resource to support future translational and hypothesis-driven research.

***Response to Reviews: 2***

*The authors have made a good job of carefully addressing my previous concerns, particularly through clarification of the methods and description of the cohort. I find the revised manuscript to be much easier to follow and believe it makes an important contribution to our understanding of cervical cancer.*
**Reply:** We sincerely thank you for the positive assessment of our revised manuscript. We greatly appreciate the constructive comments and suggestions, which have helped us substantially improve the clarity, rigor, and overall presentation of the study. We are pleased that the revisions have satisfactorily addressed your previous concerns.
