# Supplementary material for: Integrated multi-omics analysis reveals the molecular landscape and therapeutic strategies in Chinese cervical cancer patients
Source: PLoS Biol. 2026 Oct 5;24(10):e3004029. doi: 10.1371/journal.pbio.3004029 (PMC13637895; doi:10.1371/journal.pbio.3004029)
Supplement: Supplementary file 3 [file pbio.3004029.s016.docx]

***Response to Reviews: 1***

*The manuscript 'Integration of multi-omics data to unveil the molecular landscape and therapeutic strategies in cervical cancer' by Pan et al is a technically sound, high quality compendium of DNA, RNA, protein, and phosphoprotein data from a cohort of 112 Chinese patients with cervical cancer (CC), along with paired 'normal adjacent tissue' (NAT) for most of the patients. Appropriate ethical issues including informed consent are satisfactory. Strengths include a cohort of Chinese / East Asian patients of appropriate size to complement existing studies of predominantly white/North American women, the ability to compare directly to paired NAT from the same subject, and the technical quality of the DNA (WES), RNA (RNA-Seq), protein (TMT LC-MS/MS) and phosphoprotein (IMAC enriched MS/MS) data. The data appear to be appropriately available, although with different authors. It is clear that the multiomic data generated for this study represents a useful resource for cancer researchers and is likely to be mined by multiple groups for bioinformatic insights.
That said, there are several substantial issues that need to be addressed before these data can be used by others as a trusted resource.*

**Reply:** Thank you very much for your positive comments and suggestions, which greatly helped us to improve the manuscript. The manuscript has been carefully revised and point-by-point response was listed below. We hope that your comments have been addressed accurately.

*1. It is unclear who did the pathological review of the tumor specimens. The authors merely state that 'We also collected the clinical parameters of the patients, including age, tumor grade and stage (Fig 1B and S1 Table)'. Ideally, in a study of this nature, a single pathologist or team of pathologists reviews all samples to insure that there is agreement about the diagnosis of the tumor. The Supplemental Table 1 contains multiple columns labelled 'Stage' or 'Grade', and it is not clear which column represents the final consensus.*

**Reply:** Many thanks for your comments. We apologize that the pathological review and sample quality-control procedures were not sufficiently described in the original manuscript.

Before molecular profiling, three frozen sections representing the top, middle, and bottom regions of each tissue block were subjected to hematoxylin and eosin staining. All stained sections were reviewed independently by two experienced pathologists. The review included confirmation of the tumor diagnosis and histological subtype, assessment of histological grade, and estimation of tumor cellularity and necrosis. Only specimens containing at least 60% tumor cell nuclei and less than 10% necrosis were included in the multi-omics analyses.

We have provided these details in the Materials and Methods section of our revised manuscript. We have also clarified that histological diagnosis and grade were determined by pathological review, whereas tumor stage was obtained from the clinical records. In addition, we carefully revised the S1 Table. The multiple columns previously labeled “Stage” or “Grade” represented information derived from different clinical or source records but were not adequately distinguished in the original table. These columns have been renamed and reorganized, and the final variables used in all downstream analyses were explicitly labeled as “Histological grade” and “FIGO stage.” Redundant columns were removed.

*2. The Methods section is missing some key information, specifically whether the tumor tissues were subjected to either macro or micro dissection, and what the quality control criteria were for per cent cellularity, per cent tumor tissue, and per cent necrosis. Additionally, the description of protein processing for MS analysis does not specify how much tissue mass was used per extraction. These are important quality control metrics that are essential for replication of the results.*

**Reply:** Thank you for this valuable comment. We have revised the Methods section to provide detailed information regarding tissue quality assessment, tumor cellularity criteria, necrosis evaluation, and tissue input for protein extraction in our revised manuscript.

***Page 17:***

**Protein extraction**

For each protein extraction, approximately 3 mg of wet tissue was used, which yielded sufficient protein for downstream enzymatic digestion and LC-MS/MS analysis. The sample was grinded by liquid nitrogen into cell powder and then transferred to a 5-mL centrifuge tube. Four volumes of lysis buffer (8 M urea, 1% protease inhibitor cocktail, 1% phosphatase inhibitor cocktail) were added, followed by sonication on ice three times using a high-intensity ultrasonic processor (Scientz). The debris was removed by centrifugation at 12,000 g for 10 minutes at 4°C. The supernatant was then collected, and the protein concentration was measured using a BCA kit, following the manufacturer’s instructions.

For protein digestion, the sample was precipitated with TCA at 4°C for 2 hours, then washed 2-3 times with acetone and resuspended in 200 mM TEAB. Trypsin was added at a 1:50 enzyme-to-protein ratio for an overnight digestion. The protein mixture was reduced to 10 mM DTT for 1 hour at 37°C and alkylated with 20 mM IAA for 45 minutes at room temperature in the dark. A second digestion was performed by adding trypsin at a 1:100 ratio for an additional 4 hours.

***Page 19:***

**LC-MS/MS analysis**

Following phosphopeptide enrichment, peptides corresponding to 250 μg of total peptide input were subjected to high-pH reversed-phase fractionation. Each fraction was subsequently analyzed by LC-MS/MS with an absolute peptide loading amount of 1 μg per injection. Peptides were analyzed using a nanoelectrospray ionization (NSI) source followed by tandem mass spectrometry (MS/MS) on a Q Exactive HF-X (Thermo) system, coupled with an EASY-nLC 1200 UPLC. For peptide separation, the gradient was increased from 6% to 22% solvent B (0.1% formic acid in 90% acetonitrile) over 38 minutes, from 22% to 32% over 14 minutes, then ramped to 80% in 4 minutes, and held at 80% for 4 minutes. For phosphoproteome analysis, the gradient was increased from 3% to 20% solvent B over 88 minutes, from 20% to 32% over 24 minutes, ramped to 80% in 4 minutes, and held at 80% for 4 minutes.

An electrospray voltage of 2.2 kV was applied, and intact peptides were detected in the Orbitrap at a resolution of 120,000. Peptides were selected for MS/MS with a normalized collision energy (NCE) of 28, and fragments were detected at a resolution of 45,000. A data-dependent acquisition alternated between one MS scan and 15 MS/MS scans, with a dynamic exclusion of 30.0s. The automatic gain control (AGC) target was set to 5ⅹ10^4^, with an intensity threshold of 5000 and a maximum injection time of 90 ms. The first fixed mass was set at 100 *m/z*.

*3. There is no description of the procedure for obtaining NAT. How far away was the NAT from the tumor tissue? Were slides of the NAT reviewed by a pathologist to insure that the tissues were, in fact, of normal histology? Ideally, photomicrographs of representative CC and NAT stained with hematoxylin and eosin would be provided.*

**Reply:** Thanks for raising this important point. We have revised the materials and methods section with additional methodological details. These additions are intended to improve the clarity and reproducibility of our study. The revised content is provided in the materials and methods section of the revised manuscript (**S1A Fig**).

***Page 2 of supporting information:***

**S1 Fig. Mutation profiles of the CN-CC cohort, related to Fig. 1.** **(A)** Representative hematoxylin and eosin (H & E) staining images of cervical cancer tissues and matched normal adjacent tissues (NATs) used for multi-omics profiling. Scale bars, 100 μm.

***Page 15:***

**Specimen preparation**

For sample quality control of tumor samples, the following selection criteria were applied: 1) For tumor cellularity assessment, three frozen sections (top, middle, and bottom) from each tissue block were resected and subjected to H&E staining. All H & E-stained slides were scanned and analyzed pathologically; 2) Only samples with at least 60% tumor cell nuclei and less than 10% necrosis were retained; 3) Histological evaluation of all tumor samples was independently performed by two experienced pathologists.

For quality control of normal adjacent tissues (NATs), the following criteria were used for sample selection: 1) Collected from regions at least 3 cm away from the tumor margin during surgical resection; 2) Tumor cellularity analysis, three frozen sections (top, middle, and bottom) of each tissue block were resected and subjected to H & E staining. All H & E-stained slides were scanned and submitted for pathological analysis; 3) Histopathological examination confirmed the absence of tumor infiltration and high-grade intraepithelial lesions (HSIL/CIN2+); 4) The histological assessment of all tumor samples was accomplished by two experienced pathologists separately.

*Secondly, there is the usual issue with over-interpretation of results in this type of proteogenomic study, exacerbated by the lack of validation either in an independent patient cohort or in an experimental system. While these issues are usually resolved over time as other investigators build upon this work, the authors of the current study need to be transparent about the limitations of the current study, and the types of further work that would be necessary to support their claims. Following is a sample of the most problematic claims that need to be qualified.*

*1. The unsupervised hierarchical heat maps in Figure 4A do not really support the idea of 3 subtypes at the RNA, protein, and phosphoprotein levels, independent of HPV integration status. A look at the top ribbon indicates that the three subgroups are almost entirely driven by HPV integration status (non-integrated, silent, or productive) with a few discrepancies. It would have been far more interesting to do a supervised clustering based on HPV integration status and compare the resulting heatmaps.*

**Reply:** Thank you very much for your insightful feedback. Our original interpretation of Fig 4A was not sufficiently clear and might confuse the readers. Instead of emphasizing unsupervised subtype discovery, we used HPV integration status as the primary grouping variable and presented supervised heatmaps of transcriptomic, proteomic, and phosphoproteomic alterations across non-integrated, silent, and productive tumors. Samples were ordered by HPV integration status, and differentially expressed genes, proteins, and phosphosites among the three HPV-defined groups were shown after row-wise Z-score normalization. This revised figure more directly illustrates how HPV integration status is associated with coordinated multi-omics differences.

We have also revised the Results, Figure legend, and Discussion to avoid the claim that three independent molecular subtypes were identified by unsupervised clustering. The revised manuscript states that HPV integration status is associated with distinct proteogenomic patterns, rather than presenting the three groups as HPV-independent molecular subtypes. The original unsupervised clustering result, where retained, is now described as exploratory and is used only to show its concordance with HPV integration status.

***Page 8:***

HPV integration status-associated proteogenomic alterations in CN-CC

***Page 8:***

To determine whether HPV integration status was associated with coordinated molecular alterations, we performed supervised comparisons of transcriptomic, proteomic, and phosphoproteomic profiles across non-integrated, silent, and productive tumors. The supervised heatmaps revealed distinct HPV integration status-associated molecular patterns across all three omics layers included oncogenes, tumor suppressors, and tissue-enhanced genes (THE), with enrichment in key tumorigenic pathways (**Fig 4A** and **S5 Fig**). These patterns indicate that HPV integration status is a major determinant of the observed proteogenomic variation, rather than supporting three molecular subtypes independent of HPV integration status.

***Page 13:***

Integrated multi-omics analysis provides a powerful framework for delineating molecular distinctions between cervical tumors and adjacent non-tumor tissues, as well as molecular alterations associated with HPV integration status. HPV integration status was used as the primary biological grouping variable, revealing coordinated transcriptomic, proteomic, and phosphoproteomic differences among non-integrated, silent, and productive tumors.

***Page 13:***

These findings indicate that HPV integration status and viral transcriptional activity are associated with distinct proteogenomic programs, including EMT, hypoxia, interferon response, complement activation, E2F targets, MYC targets, and DNA repair pathways. These HPV-associated molecular alterations may provide mechanistic insights into cervical cancer progression and nominate candidate pathways for future therapeutic investigation.

***Page 35:***

**Fig 4. Comparative proteogenomic characteristics of cervical cancer subgroups.** **(A)** Supervised proteogenomic heatmaps of cervical tumors grouped by HPV integration status. Samples are ordered as non-integrated, silent, and productive tumors. The heatmaps showing differentially expressed transcripts, proteins, and phosphosites among the three HPV-defined groups after row-wise Z-score normalization. Top annotations indicate HPV integration status, tumor purity, and relevant clinicopathological features. The p values for categorical variables are calculated via the chi-square test. **(B)** Kaplan-Meier curves for overall survival based on RNA expression in the TCGA project cohort. n = 2309, log-rank test. **(C)** Integrated analysis of differentially activated metabolic and signaling pathways at the mRNA, protein, and phosphoprotein levels among the three CC subgroups. **(D)** Pathway scores of hypoxia, complement and E2F_target signaling pathways between subtypes and between proteomic subgroups. The line and box represent the median and upper and lower quartiles, respectively. **(E)** Diagram showing the multiomics profiles of coregulators of the EMT signaling pathway. **(F)** Identification of key regulatory factors in CC-related signaling pathways. Scatter plots showing the log2-fold change in the proteome versus mRNA between tumors and paired NATs. Different colored dots represent key regulatory factors involved in different signaling pathways. **(G)** Boxplot illustrating PLOD2 and TFRC mRNA and protein expression between subtypes as well as across proteomic subgroups. **(H)** Survival Kaplan‒Meier curves according to the PLOD2 and TFRC gene expression levels.

*2. The claims for modification of overall survival by PLOD2 status and TFRC status are over-simplified, if not outright overstated. For PLOD2, survival past 3000 days is indistinguishable for PLOD2 high versus low. For TFRC, the survival up to 2000 days is identical for TFRC high versus low. There is no acknowledgement of the clinical consequences of these survival patterns, and the overall p values are only marginally significant.*

**Reply:** Many thanks for this important and insightful comment. After re-examining the Kaplan-Meier curves, we recognized that the survival differences were not consistent across the entire follow-up period. Specifically, the PLOD2-high and PLOD2-low curves became largely indistinguishable after approximately 3000 days, whereas the TFRC-high and TFRC-low curves showed little separation before approximately 2000 days. In addition, the overall log-rank p values were only marginally significant. To address this issue, we have revised the Results and Discussion sections to provide a more cautious interpretation. We now describe these findings as modest and time-dependent survival differences observed in an exploratory analysis, rather than as strong evidence for clinically validated prognostic biomarkers. We also explicitly acknowledge the limited clinical interpretability of these survival patterns and clarify that PLOD2 and TFRC are primarily nominated as biologically relevant candidate molecules associated with E2F-related and tumor-associated pathways, rather than as established survival-stratifying markers.

***Page 13:***

Additionally, TFRC, involved in E2F signaling, and PLOD2, which facilitates EMT and metastasis under hypoxia and TGF-β1 induction, were both highly expressed in cervical tumors. The associations with overall survival were modest and time-dependent. PLOD2 expression might be not clearly distinguish long-term survival after approximately 3000 days, and TFRC expression might be not distinguish early survival risk before approximately 2000 days. These results suggested the potential biological relevance of PLOD2 and TFRC but warrant further validation in larger independent cohorts with multivariable and time-dependent survival analyses. These findings indicate that HPV integration status and viral transcriptional activity are associated with distinct proteogenomic programs, including EMT, hypoxia, interferon response, complement activation, E2F targets, MYC targets, and DNA repair pathways. These HPV-associated molecular alterations may provide mechanistic insights into cervical cancer progression and nominate candidate pathways for future therapeutic investigation.

*3. While the levels of LAMC2 and IDO1 are clearly very different between CC and NAT, it is not clear how these would be clinically useful as biomarkers, given the lack of validation in an independent cohort, and the absence of any information regarding their appearance in serum or plasma. More thought needs to be devoted to the clinical use case for candidate biomarkers.*

**Reply:** Many thanks for this important and constructive comment. To address this concern, we have performed additional validation using three independent cervical cancer transcriptomic datasets with both tumor and normal cervical samples: GSE63514, GSE9750, and GSE39001. In these analyses, CIN/LSIL/HSIL and cell-line samples were excluded, and only invasive cervical cancer and normal cervical tissue samples were compared using a two-sided Wilcoxon rank-sum test. LAMC2 was significantly upregulated in cervical cancer across all three independent cohorts. IDO1 was significantly upregulated in two datasets, and showed an increased but non-significant trend in another dataset (**S6E Fig**). These results have been provided as an independent transcriptomic validation analysis.

In addition, to explore whether these candidates are functionally related to cervical cancer biology rather than merely being differentially expressed genes, we performed an in-silico perturbation analysis using the public single-cell RNA-seq dataset GSE168652, which contains cervical tumor tissue and matched adjacent normal tissue (**S6F-S6I Fig**). Virtual knockout of LAMC2 and IDO1 induced marked downstream changes in genes related to immune infiltration, epithelial differentiation, extracellular matrix remodeling, focal adhesion, inflammatory signaling, and tumor microenvironment remodeling. For LAMC2, the top affected genes included PTPRC, MCAM, RGS1, S100A8, CD53, SERPINB3, LUM, TYROBP, C1QB, CTSS, IFNG, and CD37, with pathway enrichment involving ECM-receptor interaction (**S6F-S6G Fig**). For IDO1, the top affected genes included KRT6A, SFN, PERP, S100A2, S100A9, IFI27, LY6D, SLPI, SERPINB3, SPARCL1, KRT17, KRT19, and SDC1, with pathway enrichment involving ECM-receptor interaction, focal adhesion, and regulation of the actin cytoskeleton (**S6H-S6I Fig**). Notably, several downstream genes, such as S100A8 and SERPINB3, have been linked to cervical squamous cell carcinoma progression or prognosis in previous studies. These findings further support a biologically meaningful tissue-level role for the LAMC2/IDO1-associated marker program.

Third, we have also revised the manuscript to clarify the intended clinical use case of these molecules. In particular, LAMC2 may reflect extracellular matrix remodeling, epithelial-mesenchymal transition, and invasive tumor biology, supporting its potential use in tissue-based molecular characterization or therapeutic hypothesis generation. IDO1, by contrast, is more closely related to immune regulation and may be more relevant for immune microenvironment classification or immunotherapy-oriented stratification rather than general cancer detection.

Importantly, we further acknowledge that our current study did not evaluate serum or plasma samples. Therefore, we have added a limitation stating that the current study does not provide evidence for serum or plasma detectability of LAMC2 or IDO1. Future studies aim to develop them as circulating biomarkers, targeted proteomic assays or ELISA-based measurements in serum or plasma from independent patient cohorts are needed.

***Page 7 of supporting information:***

**S6 Fig.** **Identification of early proteomic detection and therapeutic biomarkers, related to Fig. 5. (A-B)** Relative protein abundance and mRNA expression of STAT1 and PARP1 between tumors and NATs (Wilcoxon rank-sum test) as well as across cervical cancer subgroups (Kruskal-Wallis test). **(C)** Relative protein abundance and mRNA expression of MKI67 between tumors and NATs (Wilcoxon rank-sum test) as well as across cervical cancer subgroups (Kruskal-Wallis test). The line and box represent the median and upper and lower quartiles, respectively. **(D)** Spearman correlation between the mRNA expression and protein abundance of LAMC2 and IDO1. **(E)** LAMC2 and IDO1 expression across three GEO cohorts. **(F, H)** The top 20 genes with significant changes after performing gene perturbation analysis (LAMC2 or IDO1) using scTenifoldKnk on the top 500 highly variable genes. The length of each bar represents the log Fold Change (logFC) of the corresponding gene, and the color gradient from light yellow to dark purple indicates an increasing degree of change. **(G, I)** A ridge plot of GSEA functional enrichment analysis based on genes ranked by the standardized perturbation strength (Z-value). Each ridge corresponds to a significantly enriched KEGG pathway. The x-axis represents the gene perturbation strength (Z-value), indicating the extent to which genes are affected after virtual knockout of the target gene (LAMC2 or IDO1).

***Page 9:***

Integrated multi-omics analysis nominated 12 candidate tissue-associated proteins that were differentially expressed between cervical tumors and NATs and may reflect tumor-associated biological programs (**Fig 5A**). Consistent with prior evidence linking elevated PARP1 mRNA to poor prognosis, we observed significant upregulation of STAT1 and PARP1 at both mRNA and protein levels (**S6A-S6B Fig**).

***Page 14:***

Elevated expression of LAMC2 is established as a promoter of tumor invasion, metastasis, angiogenesis, and reduced apoptosis. Furthermore, genes overexpressed in the high-LAMC2 subgroup of cervical cancer were significantly enriched in key oncogenic pathways, including focal adhesion, PI3K-AKT, HPV infection, and ECM-receptor interaction (**Fig 5I**). Concurrently, IDO1 contributes to immune escape in cervical squamous cell carcinoma by mediating immune tolerance and suppressing autoimmunity. These collective findings nominate LAMC2 as a candidate tissue-associated marker of extracellular matrix remodeling and invasive tumor biology. However, further validation in independent cohorts, preferably using standardized tissue-based assays such as immunohistochemistry, will be required to determine whether LAMC2 can support pathological risk stratification or therapeutic development.

*4. The entire immune landscape section is very confusing due to the absence of key details. The authors claim 6 immune subtypes without providing a consensus clustering matrix (as in Figure S5) to justify 6 distinct subtypes, and they never really describe what is driving the 6 subtypes. They immediately go to lumping the 6 subtypes into 3: 'immune hot, immune warm, and immune cold', and all subsequent comparisons are between hot, warm, and cold. The bar graphs in 6D, 6E, and 6G appear to support 3 subtypes, not 6, and the K-M plot in Figure 6H shows a very clear difference in survival which warrants further analysis and experimentation. However, the rather arbitrary 6 immune subtypes merely confuses the analysis.*

**Reply:** Many thanks for your comments. Our descriptions in the original manuscript might confuse the readers. To address this concern, we have revised the immune landscape section. We now use the three immune phenotypes (immune-hot, immune-warm, and immune-cold) as the primary framework for the main text and figures. The former six-cluster classification is no longer presented as the central conclusion. Instead, where retained, it is described only as an exploratory substructure within the three broader immune phenotypes. We have also revised the Results, figure legend, and Discussion to remove wording that implies that six immune subtypes represent robust, clinically independent categories.

In the revised manuscript, the immune-hot phenotype is defined by higher ESTIMATE immune scores, increased infiltration of effector memory CD4^+^ T cells and macrophages, elevated expression of immune checkpoint molecules and cytotoxic markers, lower TIDE scores, and better overall survival. The immune-cold phenotype shows the opposite pattern, whereas the immune-warm phenotype displays intermediate immune infiltration and pathway activity. This revised organization is more consistent with the patterns shown in Fig 6D, 6E, 6G, and 6H and provides a clearer biological interpretation of the immune landscape.

The survival difference observed in Fig 6H is potentially important. We have therefore revised the text to emphasize this finding more directly and to state that the association between immune phenotype and overall survival warrants further validation in independent cohorts and, where possible, functional or clinical follow-up studies.

***Page 10:***

We characterized the immune infiltration landscape of cervical cancer using xCell deconvolution and ESTIMATE-based immune scoring. Based on the overall immune and stromal infiltration patterns, tumors were classified into three major immune phenotypes: immune-hot, immune-warm, and immune-cold (**Fig 6A**). The immune-hot phenotype showed higher immune infiltration, whereas the immune-cold phenotype showed markedly reduced immune and stromal signals, with the immune-warm phenotype displaying intermediate features. This three-phenotype framework was used for the main downstream analyses because it provided the clearest biological and clinical interpretation of the immune landscape (**Fig 6A-6B** and **S7A-S7D Fig**).

***Page 11:***

Compared with the immune-warm and immune-cold phenotypes, immune-hot tumors exhibited significantly higher ESTIMATE immune scores and enhanced infiltration of effector memory CD4^+^ T cells and macrophages **(Fig 6D** and **Fig 6F-6G**). Tumor mutational burden and immune infiltration also differed across these three immune phenotypes (**Fig 6E**). Importantly, patients with immune-hot tumors showed more favorable overall survival than those with immune-warm or immune-cold tumors (**Fig 6H**). TIDE analysis further showed lower immune evasion scores in the immune-hot phenotype, suggesting that these tumors may be more immunologically active and potentially more responsive to immune checkpoint blockade (**Fig 6I**). These findings support the biological and clinical relevance of the three immune phenotypes, although further validation in independent cohorts is required.

***Page 12:***

Multi-omics analyses revealed key oncogenic drivers (e.g., PIK3CA, EP300, MPRIP) and pathway activation, with LAMC2 emerging as a promising diagnostic and therapeutic target. Immune profiling delineated three major tumor microenvironment phenotypes (immune-hot, immune-warm, and immune-cold) with distinct immune infiltration, checkpoint expression, TIDE scores, and survival patterns. Our findings elucidate genomic, transcriptomic, and proteomic disruptions and their regulatory relationships, underscoring the value of proteogenomic integration in uncovering novel cancer biology and therapeutic hypotheses for CN-CC patients.

*Minor typo in the legend for Figure 5; between is missing the 'b' [etween].*

**Reply:** Thanks for your comments. We have accepted the suggestion and corrected these issues according your suggestions. The manuscript has been greatly improved by addressing your valuable comments.

***Response to Reviews: 2***

*This manuscript describes the multi-omics analysis of a Chinese cervical cancer cohort, which complements notable existing studies from European, African and US-based cohorts and therefore has the potential to make an important contribution to our understanding of cervical cancer biology and its similarities across different ethnic groups. A particular strength of this study, which sets it apart from the aforementioned studies is the application of global proteomics to interrogate differential protein expression and activation states on a much larger (and unbiased) scale than the RPPA approach used by TCGA, for example. I do have some concerns the regarding clarity of reporting and the methodology employed.*

**Reply:** Thank you very much for your positive comments and suggestions, which greatly helped us to improve the manuscript. The manuscript has been carefully revised and point-by-point response was listed below. We hope that your comments have been addressed accurately.

*The study relies heavily on comparisons between three subgroups defined by the authors based on the physical status of the HPV genome and on HPV transcriptional activity: HPV-integrated with evidence of HPV gene expression ("productive"); HPV-integrated without evidence of HPV gene expression ("silent") and non-integrated subgroups. However, insufficient detail is provided in the methods for the reader to understand how these groups were defined and to give confidence that samples have been accurately attributed to these groups. The methods section lacks any description of how HPV typing and assessment of HPV gene expression was conducted. In the description of alignment for the WES and RNA-seq data, only alignment to the human reference genome is mentioned but presumably, HPV genomes were also included, either appended to GRCh38 to make a combined human/viral reference, or unaligned reads from the BAM file generated by alignment to GRCh38 were taken and aligned to HPV genomes? If not, how were samples classified as transcriptionally active or silent, and where does the evidence supporting integrated versus non-integrated status for each sample come from? This needs to be detailed for both WES and RNA-seq data (including information on the source of the HPV genomes, accession numbers etc). Note that whole exome sequencing is not a reliable method (at least when used in isolation) by which to determine HPV integration status. Whole genome sequencing (data now available for TCGA's cohort, so deriving accurate integration status for TCGA samples from this could be of use to the authors) is much more reliable for detecting and mapping HPV integration. In lieu of this, capturing HPV genome sequences in addition to the human exome would enable more reliable detection of integration than relying on exome data alone.*
**Reply:** Many thanks for this important and constructive comments. We have accepted the suggestions and provided detail methods in our revised manuscript.

In the present study, the HPV genotype and HPV integration/transcriptional activity annotations were not inferred de novo from WES data alone. Instead, these annotations were obtained from our previously published study (*Fan et al., Cell Genomics, 2023*), in which HPV typing, DNA-level HPV integration, and RNA-level HPV transcriptional activity were systematically characterized using HPV-focused multi-omics analyses. In that study, HPV integration status was determined based on virus–host chimeric evidence at the DNA level, while transcriptionally active HPV integration was assessed using RNA-seq evidence for HPV transcripts and virus-host fusion transcripts.

Accordingly, in the current manuscript, samples were classified into three groups according to the previously established annotations: HPV-integrated tumors with evidence of HPV gene expression and/or virus-host fusion transcripts were defined as the productive HPV integration group; HPV-integrated tumors without detectable evidence of HPV transcriptional activity were defined as the silent HPV integration group; and tumors without evidence of HPV integration were classified as the non-integrated group. We have now clarified that WES data in the present study were used for human genomic analyses and were not used in isolation to determine HPV integration status. We fully agree with the reviewer that WES alone is not sufficiently reliable for accurate HPV integration detection, and this point has now been explicitly stated in the revised Methods section.

To address the reviewer’s concern, we have revised the Materials and Methods section by adding a dedicated description of HPV typing, HPV genome reference sources, assessment of HPV transcriptional activity, and subgroup definition criteria. We have also cited the original Cell Genomics study from which the HPV genotype and integration annotations were derived, and provided the accession information for the HPV reference genomes used in the original analysis. These revisions can be found in the revised manuscript.

***Page 15:***

**HPV typing and classification of HPV integration status**

HPV genotype information and HPV integration/transcriptional activity annotations were obtained from our previously published study, Multi-omics characterization of silent and productive HPV integration in cervical cancer (*Fan et al., Cell Genomics, 2023*). Briefly, For VCS data, cleaned reads were aligned to the human-HPV reference using BWA-MEM and deduplicated with Picard. HPV genotypes were identified using a mean viral genome read depth ≥10, and SurVirus-detected integration breakpoints supported by ≥10 reads were retained. For bulk RNA-seq, reads were aligned using STAR, HPV abundance was quantified with Subread, and HPV types with CPM ≥1 were retained. Virus-host fusion transcripts were identified from STAR chimeric alignments, requiring ≥10 supporting reads. HPV typing and HPV integration analyses were performed using HPV-aware sequencing analyses with HPV reference genomes obtained from public databases. DNA-level HPV integration was determined based on virus-host chimeric evidence, whereas RNA-level transcriptional activity was assessed by detecting HPV transcripts and virus-host fusion transcripts from RNA-seq data. Tumors with DNA-level HPV integration and detectable HPV transcriptional activity were classified as having productive HPV integration; tumors with DNA-level HPV integration but without detectable HPV transcriptional activity were classified as having silent HPV integration; and tumors without evidence of HPV integration were classified as non-integrated. In the present study, WES data were used for human genomic alteration analysis and were not used alone to infer HPV integration status.

*Related to the above point, it is surprising to see so many cervical SCCs in this cohort in which HPV gene expression appears to have been undetectable using RNA sequencing. This is a significant difference from TCGA's cohort, from the cohort investigated by Ojesina et al (cited as reference 20) and from CGCI's Uganda cohort (*[*https://doi.org/10.1038/s41588-020-0673-7*](https://doi.org/10.1038/s41588-020-0673-7)*). In the absence of any information in the methods section about how HPV reads were identified and quantified, it is impossible for me to assess what may explain this unexpected finding. Was a cutoff for HPV transcript reads defined, below which a sample was defined as silent?*

**Reply:** Many thanks for your comments. HPV genotype, HPV integration status, and HPV transcriptional activity were derived from our laboratory’s previously published study (*Fan et al., Cell Genomics, 2023*), in which HPV-aware analyses were performed to characterize both DNA-level HPV integration and RNA-level viral transcriptional activity. In that study, HPV-derived reads and virus–host chimeric reads were identified using HPV reference genomes in addition to the human reference genome, and HPV transcriptional activity was assessed based on detectable HPV transcripts and virus–host fusion transcripts. The distinction between productive and silent HPV integration was therefore based on predefined viral transcriptional evidence rather than on the absence of reads in human-only alignments. Fan et al. reported that productive HPV integration was associated with higher E6/E7 protein expression and more aggressive tumor features, supporting the biological relevance of this classification.

We have further revised the Methods section to include the source of HPV genotype and integration annotations, the HPV reference genome information, the read-identification strategy, and the cutoff used to define HPV transcriptional activity. These additions are intended to improve transparency and reproducibility and to allow readers to better interpret the difference between our cohort and previously reported cohorts. The corresponding revisions have been added to the Materials and Methods section of the revised manuscript on ***page 15***.

*The schematic diagram in Figure 1A is helpful in explaining how many cancer samples were analysed in each of the different experiments (112 for proteomics, and subsets of 97 for RNA-seq (+ 97 NAT) and 98 for whole exome seq (+81 NAT). But the text does not correspond very clearly to this, so for example, in the Results section "Thus, we conducted genomic analysis on paired tumors and adjacent tissues from 98 CC patients" Should this not read "paired tumors and adjacent tissues from 81 CC patients, and tumor-only from an additional 17?" If so, it is also essential to clearly describe how somatic variants were called from the 17 samples for which no paired normal tissue was taken. Or was DNA isolated from blood, or NAT from the proteomics study (for which NAT was available for all 112 cases) to get the germline exome sequence data?*

**Reply:** Thanks for this careful and important comment. In the revised manuscript, we have corrected this description to state that WES was performed on 98 tumor samples, among which 81 tumors had matched adjacent normal tissues for paired tumor–normal somatic variant calling, whereas 17 tumors did not have matched normal WES data and were therefore analyzed using a tumor-only strategy with a cohort-specific normal baseline/panel of normals.

Specifically, for the 81 tumors with matched normal tissues, somatic SNVs and indels were identified using a conventional paired tumor–normal approach. For the remaining 17 tumor-only WES samples, no blood-derived germline WES or matched normal WES data were available. Therefore, to reduce contamination from germline variants and recurrent sequencing artifacts, we constructed a cohort-specific normal baseline using all available normal WES samples sequenced in this study under the same experimental and bioinformatic conditions. Variants detected in the 17 tumor-only samples were filtered against this normal baseline, together with additional quality-control filters and population germline variant databases where applicable. This strategy is conceptually consistent with the panel-of-normals approach recommended for tumor-only somatic variant analysis, in which recurrent variants observed across normal samples are used to remove likely germline variants and platform-specific artifacts. The GATK best-practice documentation describes that a panel of normals for short variants is generated by calling variants in multiple normal samples and combining recurrent sites into a sites-only VCF for subsequent filtering, and Mutect2 also supports tumor-only analysis with a panel of normals and germline-resource filtering.

Tumor-only somatic variant calling is less definitive than paired tumor-normal analysis, because private germline variants cannot be completely removed in the absence of a patient-matched normal sample. Therefore, we have now explicitly described this limitation in the revised Methods and Discussion sections. In addition, we have added methodological details describing how somatic variants were called in the 17 tumor-only samples, including construction of the normal baseline, filtering criteria, and quality-control procedures. These revisions can be found in the Materials and Methods section of the revised manuscript.

***Page 19:***

**Somatic variant calling for paired and tumor-only WES samples**

WES data were available for 98 tumor samples. Among them, 81 tumors had matched adjacent normal tissue WES data and were analyzed using a paired tumor-normal somatic variant-calling strategy. The remaining 17 tumors did not have matched normal WES data and were analyzed using a tumor-only strategy. For these tumor-only samples, we constructed a cohort-specific normal baseline from all available normal WES samples generated in this study using the same sequencing platform, capture kit, and bioinformatic pipeline. Recurrent variants detected in normal samples were used to define a normal baseline and were excluded from tumor-only variant calls. Candidate variants were further filtered according to sequencing depth, variant allele fraction, mapping quality, strand bias, read-position bias, and population allele frequency in public germline databases, where applicable.

Variants retained after these filtering steps were considered putative somatic variants for the 17 tumor-only samples. Because matched germline DNA was unavailable for these cases, we acknowledge that residual private germline variants cannot be completely excluded. Therefore, tumor-only variant calls were interpreted with caution and were subjected to more stringent filtering than paired tumor–normal calls.

*Why are the sample numbers different between Figure 1G (n=98) and Figure 1H (n=81)? Why could HRD scores not be defined for the entire WES cohort? Again, essential methodological detail is missing - there is no description of how HRD scores were calculated in the methods section.*

**Reply:** We thank the reviewer for pointing out this important omission. The difference in sample size is due to the different data requirements of the two analyses. Fig 1G was based on coding mutation rates and APOBEC-associated mutational signatures, which could be evaluated in all 98 tumor WES samples that passed quality control. In contrast, Fig 1H was based on genomic scar-based HRD scores, which require reliable allele-specific copy-number and loss-of-heterozygosity information from matched tumor-normal WES data. Among the 98 WES tumor samples, 81 had matched adjacent normal tissue WES data suitable for tumor-normal allele-specific copy-number analysis, whereas 17 samples were tumor-only WES cases. Therefore, Fig 1H was restricted to the 81 paired tumor-normal WES samples.

To address this issue, we have revised the Results section, Fig 1 legend, and Materials and methods section. We now explicitly state that Fig 1G includes all 98 WES tumors, whereas Fig 1H includes only the 81 paired tumor-normal WES samples. We have also added a new Methods subsection describing HRD score estimation.

***Page 15:***

**HRD score estimation**

Allele-specific copy-number profiles were inferred from paired tumor-normal WES BAM files using Sequenza and then used as input for scarHRD. Homologous recombination deficiency (HRD) scores were calculated using the scarHRD package. Briefly, allele-specific copy-number profiles were first generated from paired tumor-normal WES data and then used as input for scarHRD. scarHRD estimates three genomic scar components: loss of heterozygosity (HRD-LOH), telomeric allelic imbalance (TAI), and large-scale state transitions (LST). The final HRD score was defined as the sum of the LOH, TAI, and LST scores. Samples lacking matched normal WES data were excluded from HRD score estimation because tumor-only sequencing does not provide a reliable germline baseline for distinguishing somatic allelic imbalance from inherited copy-number variation or mapping artifacts. Therefore, HRD scores were calculated for 81 paired tumor-normal WES samples, whereas the 17 tumor-only WES samples were not assigned HRD scores. Samples with HRD scores >30 were considered to have moderate-to-high HRD in this analysis.

*Mutational signature analysis - although the authors state in the methods that "The number of signatures was determined by choosing the reflection point in the curves of explained variance and residual sum of squares (RSS)", which is a reasonable approach, it looks like they have overestimated the number of signatures present. For example their signature S5 clearly looks to be a combination of S1 and S3 (SBS2 and SBS13 respectively), rather than being a separate signature. It would be worth looking again at this analysis and potentially restricting to signatures that have been reported to be present in cervical cancer (e.g. by reference to COSMIC (*[*https://cancer.sanger.ac.uk/signatures/*](https://cancer.sanger.ac.uk/signatures/)*) and Signal (*[*https://signal.mutationalsignatures.com/*](https://signal.mutationalsignatures.com/)*).*

**Reply:** Many thanks for this important and constructive comment. We initially selected the number of signatures based on the inflection point of the explained variance and residual sum of squares curves. To address this concern, we have re-evaluated the mutational signature analysis by incorporating COSMIC and Signal reference signature databases. First, each de novo signature was compared with COSMIC/Signal SBS reference signatures using cosine similarity (**S2 Table**). This analysis showed that S1 closely matched SBS2 (cosine similarity = 0.971) and S3 closely matched SBS13 (cosine similarity = 0.931), both of which are APOBEC-associated signatures. In contrast, S5 showed only moderate similarity to APOBEC- or polymerase-associated reference signatures, including SBS2/SBS10b, and was therefore not retained as an independent mutational process. We now interpret S5 as an over-decomposed or ambiguous APOBEC/polymerase-like component rather than as a separate biological signature.

Second, we revised the signature interpretation by merging or filtering de novo components according to COSMIC/Signal annotation. APOBEC-associated components were merged into a single APOBEC burden defined as the combined contribution of SBS2 and SBS13 (**Fig 1F** and **S1G Fig**). Components mapping to the same DNA repair-related process, including SBS6-, SBS20-, and SBS26-like signatures, were interpreted conservatively as DNA repair-associated signatures detected in subsets of tumors rather than as multiple independent processes (**Fig 1F**). S2, which matched SBS52, was excluded from biological interpretation because SBS52 is annotated as an artefact-related signature. Low-similarity or unstable components were not interpreted as independent biological signatures.

Third, we revised the downstream analyses accordingly. In the revised manuscript, APOBEC burden is defined as the combined contribution of SBS2 and SBS13 rather than as individual de novo signatures (**S1H Fig**). We repeated the comparisons of APOBEC burden across HPV integration groups and its associations with coding mutation burden and HRD score using this revised definition (**S1I-S1J Fig**). These analyses retained the main conclusion that APOBEC-associated mutagenesis is enriched in HPV-integrated tumors and associated with increased mutation burden, while avoiding overinterpretation of individual de novo components.

The previous statement that 13 distinct mutational signatures were identified has been removed or rephrased as an exploratory de novo extraction result. The revised manuscript now emphasizes the COSMIC/Signal reference-based mutational signature landscape, particularly the dominant contribution of SBS2/SBS13 APOBEC mutagenesis in cervical cancer.

***Page 6:***

Initial de novo extraction identified 13 mutational patterns across the cohort (**S1C-S1F Fig**). However, comparison with COSMIC SBS signatures revealed that several extracted signatures showed substantial overlap with known mutational processes (**S2 Table**). In particular, the S5 signature demonstrated limited similarity to SBS10b and lacked independent biological relevance, whereas its mutation spectrum closely resembled APOBEC-associated SBS2 and SBS13 (**Fig 1F**). Therefore, S5 was considered a composite APOBEC-related pattern rather than an independent mutational process. Mutational processes were consolidated into four major biological categories, including APOBEC-associated, age/background, DNA repair-associated, and low-confidence/artifactual signatures (**S1H-S1J Fig**).

**S2 Table**. The relationship between CN-CC identified mutational signature and COSMIC curated mutational signatures.

| **Signatures** | **COSMIC** **signatures (Cluster)** | **COSMIC signatures (Cosine similarity)** | **Similarity** | **Comments**  **(Cluster / Cosine similarity)** |
| --- | --- | --- | --- | --- |
| S1 | SBS2 | SBS2 | 0.971 | APOBEC activity |
| S2 | SBS52 | SBS52 | 0.940 | Sequencing artifact (blacklisted cancer samples for poor quality) |
| S3 | SBS13 | SBS13 | 0.931 | APOBEC activity |
| S4 | SBS20 | SBS20 | 0.870 | POLD1 mutations and DNA mismatch repair |
| S5 | SBS10b | SBS2 | 0.618 | POLE exonuclease domain mutation / APOBEC activity |
| S6 | SBS6 | SBS6 | 0.922 | DNA mismatch repair and microsatellite instability (MSI) |
| S7 | SBS1 | SBS1 | 0.880 | Spontaneous deamination of 5-methylcytosine |
| S8 | SBS6 | SBS6 | 0.798 | DNA mismatch repair and microsatellite instability (MSI) |
| S9 | SBS7b, SBS30 | SBS7b | 0.590 | inactivating mutations in NTHL1 / UV light exposure |
| S10 | SBS58 | SBS2 | 0.519 | APOBEC activity |
| S11 | SBS58 | SBS7a | 0.434 | APOBEC activity |
| S12 | SBS33, SBS54 | SBS26 | 0.465 | DNA mismatch repair and microsatellite instability (MSI) |
| S13 | SBS5, SBS92 | SBS5 | 0.546 | Unknown |

***Page 30:***

**Fig 1. Multiomics landscape of CN-CC samples.** **(A)** Overview of the experimental design and number of samples for proteomics, phosphoproteomics, WES, and RNA-seq analyses. The gray blocks in the schematic represent the missing data. The annotations to the right indicate samples in each category (bottom panel). WES, whole-exome sequencing. **(B)** Clinical information on the CN-CC samples. **(C)** Genetic profiles and associated clinicopathologic features of all 98 CC patients. **(D)** Comparisons of the top 30 significantly mutated genes between the CN-CC cohort and the TCGA CESC cohort. **(E)** Lollipop plot visualizing all protein-altering somatic mutations detected in this cohort. The size of a lollipop represents the number of samples with corresponding mutations, and the color represents a specific type of mutation. **(F)** Unsupervised clustering results between the COSMIC signatures and 13 signatures of the CN-CC cohort. **(G)** Differences in coding mutation rates across sample types exhibiting low (≤ 0.4) and high (> 0.4) APOBEC signatures (n = 98 WES tumors). **(H)** Differences in coding HRD scores across sample types exhibiting low (≤ 0.4) and high (> 0.4) APOBEC signatures (n = 81 paired tumor-normal WES samples).

***Page 2 of supporting information:***

**S1 Fig. Mutation profiles of the CN-CC cohort, related to Fig. 1.** **(A)** Representative hematoxylin and eosin (H&E) staining images of cervical cancer tissues and matched normal adjacent tissues (NATs) used for multi-omics profiling. Scale bars, 100 μm. **(B)** Distribution of mutation burdens in each cancer type. Each dot corresponds to the mutation burden calculated from a tumor-normal pair. **(C)** Lollipop plot visualizing all protein-altering somatic mutations detected in this cohort. The size of a lollipop represents the number of samples with corresponding mutations, and the color represents a specific type of mutation. EP300 gene. **(D)** Four clusters identified by NMF. **(E)** Upper, residual sum of squares (RSS) of different signature number selections. The percentage of variance explained by different signature number selections is lower. **(F)** Thirteen mutation signatures were detected in the CN-CCs. **(G)** Unsupervised clustering results between the COSMIC signatures and 13 signatures of the CN-CC cohort. **(H)** Constrained refitting of reconstructed sample spectra. **(I-J)** Contributions of 13 mutation signatures in different stages and sample types. * *p* < 0.05.

*The introduction is very brief and while TCGA's 2017 landscape study is mentioned, it does not provide a strong overview of the context in which the present manuscript should be viewed. Key cervical cancer genomics studies such as Ojesina et al Nature 2014 (which preceded TCGA's 2017 publication) should at least be discussed at this point. The decision to highlight SHKBP1, ERBB3, and TGFBR2 from TCGA's publication is also surprising, given that these are not among the most common somatic alterations detected in that study, or in the previous study from Ojesina et al. Driver mutations in PIK3CA and EP300 for example, were reported at much higher frequencies in both these studies. The multi-omics analysis of cervical squamous cell carcinoma conducted by Chakravarthy et al (Chakravarthy et al Nat Comms 2022 (*[*https://doi.org/10.1038/s41467-022-33544-x*](https://doi.org/10.1038/s41467-022-33544-x)*) is also highly relevant to the work conducted here and made observations that are relevant to this study e.g. concerning EMT, Hippo/YAP signalling and the immune microenvironment. The authors should make more effort to consider their findings in the context of these previous studies and to highlight similarities and differences. This is particularly important given the fact that as they correctly point out, Asian patients have been underrepresented in these studies, so it would be very useful to understand whether there are potential differences by ethnicity. The authors might consider for example, allocating their samples to the gene expression-based clusters identified by TCGA and by Chakravarthy et al. Given that these clusters were associated with differences in survival, it would be very interesting to see whether these differences are apparent in this cohort of Asian patients. Finally, given the rationale for identifying markers that could predict ICI response, it would also be useful to include some background context on the use of ICI in cervical cancer.*

**Reply:** Thanks for this important and constructive comment. We have therefore revised the Introduction to provide a more balanced overview of the cervical cancer molecular landscape, highlighting recurrent drivers such as PIK3CA, EP300, FBXW7, PTEN, STK11, MAPK1, HLA-B, and NFE2L2, while also acknowledging the novel genes identified by TCGA.

Specifically, we have expanded the Introduction to discuss the landmark study by Ojesina et al., which preceded TCGA and identified recurrent genomic alterations in cervical carcinomas, including EP300, FBXW7, MAPK1, HLA-B, NFE2L2, TP53, and ERBB2. We also revised the discussion of the TCGA 2017 study to include APOBEC mutagenesis, HPV integration, recurrent driver mutations, CD274/PD-L1 and PDCD1LG2/PD-L2 amplifications, and integrative molecular subgroups. In addition, we added discussion of the integrated multi-omics analysis by Chakravarthy et al., which identified two conserved cervical squamous cell carcinoma subtypes with prognostic significance and differences in EMT, Hippo/YAP signaling, immune checkpoint expression, and the tumor immune microenvironment.

Finally, we expanded the Introduction to include the clinical context of immune checkpoint inhibition in cervical cancer, including the established activity of PD-1 blockade in persistent, recurrent, or metastatic cervical cancer and the need for biomarkers that more accurately identify immune-active tumors.

***Page 3:***

**Introduction**

Cervical cancer (CC) remains one of the most common malignancies among women worldwide, with persistent human papillomavirus (HPV) infection representing the primary etiological factor. Although HPV vaccination and screening programs have reduced disease incidence in some regions, cervical cancer continues to cause substantial morbidity and mortality, particularly in low- and middle-income countries where access to preventive and therapeutic strategies remains limited. Genomic, transcriptomic, epigenomic, and proteomic studies have therefore been essential for defining recurrent driver events, molecular subtypes, and therapeutically relevant vulnerabilities in this disease.

Comprehensive genomic studies have substantially advanced our understanding of cervical cancer biology. It has been reported that recurrent genomic alterations in cervical cancer frequently involve key driver genes such as PIK3CA, EP300, and FBXW7, together with broader dysregulation of chromatin remodeling and oncogenic signaling pathways. Subsequently, The Cancer Genome Atlas (TCGA) study integrated genomic, transcriptomic, epigenomic, and proteomic datasets from cervical carcinomas and defined recurrent molecular alterations involving PI3K/MAPK signaling, immune-related pathways, and HPV-associated genomic remodeling. These studies established the molecular landscape of cervical cancer; however, most patients included in these cohorts were of European or North American ancestry, and Asian populations remain underrepresented.

Beyond genomic alterations, recent multi-omics studies have highlighted the importance of transcriptional and microenvironmental heterogeneity in cervical cancer. Previous multi-omics analyses have shown that cervical cancer can be stratified into molecular subtypes with distinct EMT-related, Hippo/YAP-associated, and immune microenvironmental features. These findings suggest that cervical cancer heterogeneity cannot be fully explained by genomic alterations alone and emphasize the importance of integrated multi-dimensional profiling.

Despite these advances, comprehensive multi-omics characterization of cervical cancer in Asian populations remains limited. Given potential differences in genetic background, HPV genotype distribution, environmental exposure, and immune landscape, whether previously defined molecular subtypes and immune phenotypes are applicable to Asian patients remains unclear. Therefore, large-scale multi-omics profiling of Asian cervical cancer cohorts is essential to define population-specific molecular characteristics and identify clinically actionable biomarkers.

Here, we performed integrated proteogenomic analyses—spanning genomics, transcriptomics, proteomics, and phosphoproteomics—on matched tumor and adjacent normal tissues from 112 Chinese cervical cancer patients. Our study emphasizes clinically actionable insights derived from proteomic and post-translational modification profiles, capturing functional consequences of genomic alterations. This approach revealed distinct disease subgroups with prognostically relevant phenotypes, driven by underlying genetic variations. Additionally, we characterized the tumor immune microenvironment, identifying potential predictive markers and therapeutic targets to guide immune checkpoint inhibitor (ICI) selection. Collectively, this work provides a comprehensive multi-omics resource that delineates the pathogenic mechanisms of CC and proposes candidate biomarkers, thereby supporting future efforts in precision diagnostics and targeted therapy development for this population.

*Initial description of the cohort: I notice from Table S1 that all tumours analysed are squamous cell carcinomas - it would be useful to state this, either in the introduction or at the beginning of the results section.*

**Reply:** Many thanks for this helpful comment. We would like to clarify that the cohort was not composed exclusively of squamous cell carcinomas. As shown in Table S1, the cohort included both squamous cell carcinomas and adenocarcinomas. The previous version of the manuscript did not explicitly state this information in the Introduction or at the beginning of the Results section, which may have caused confusion. We have provided details of this issue in the revised manuscript.

***Page 5:***

To comprehensively characterize the molecular landscape of Chinese cervical cancer (CN-CC), we curated high-quality tumor and adjacent normal tissue (NAT) samples from 112 patients (**Fig 1A** and **S1 Table**). The cohort included both squamous cell carcinomas and adenocarcinomas, with squamous cell carcinoma representing the predominant histological subtype ([n = 108, 96.4%] squamous cell carcinomas; [n = 4, 3.6%] adenocarcinomas).

*I would advise against use of the term 'productive' to describe tumours in which HPV gene expression is detected, as this will cause confusion with the term productive HPV infection - used to describe the normal life cycle of the virus which results in the production of HPV virions. A suitable alternative might be 'transcriptionally active', or simply 'active'.*

**Reply:** Thanks for your suggestion. We have accepted the suggestion and corrected these issues according your suggestions. The manuscript has been greatly improved by addressing your valuable comments.

*For the statement, "PIK3CA mutations were predominantly localized to the helical domain (E542K, E545K), a pattern consistent with bladder cancer and HPV-positive head and neck squamous cell carcinomas, but distinct from breast cancer (Fig 1E). These substitutions are potentially linked to APOBEC-mediated mutagenesis…." The correct primary reference to cite for this is Henderson et al Cell Reports 2014 (*[*https://doi.org/10.1016/j.celrep.2014.05.012*](https://doi.org/10.1016/j.celrep.2014.05.012)*).*

**Reply:** Thanks for your suggestion. We have accepted the suggestion and corrected these issues according your suggestions. The manuscript has been greatly improved by addressing your valuable comments.

*Since Figure 5J represents findings from protein-level data, protein names should be used (i.e. PIK3CA encodes the p110-alpha catalytic subunit of PI3K - is this the protein that the authors have found to be phosphorylated, as suggested in the schematic diagram? Phosphorylation of p110-alpha is not a well described regulatory mechanism, rather it is recruited to activated RTKs via binding of its regulatory subunit (p85) to phosphotyrosine motifs on the receptors. If the phospho-proteomics data do not identify PI3K phosphorylation but rather an inferred increase in activation based on increased AKT phosphorylation, the diagram should be modified accordingly.*
**Reply:** Thank you for this important and technically insightful comment. In response, we have revised Fig 5J accordingly. Specifically, we used protein-level nomenclature throughout the schematic (with gene symbols retained where appropriate for clarity), and removed the implication that p110α/PIK3CA is directly activated by phosphorylation. Instead, the revised figure presents a more conservative and biologically appropriate model in which increased LAMC2/laminin-332 abundance is associated with extracellular matrix remodeling and integrin receptor engagement, converging on focal adhesion remodeling and downstream PI3K-AKT-related signaling. In the revised model, PI3K pathway activation is interpreted as an inferred downstream signaling state supported by integrated pathway enrichment and downstream phosphoproteomic changes, particularly AKT-related signaling, rather than as direct evidence for activating phosphorylation of p110α.

Accordingly, the revised Fig. 5J now emphasizes a LAMC2-associated ECM-receptor interaction model involving laminin-332 components, integrin receptor axes, focal adhesion kinases/scaffold proteins, and downstream PI3K-AKT and MAPK/EMT-related signaling outputs. We believe this revision more accurately reflects the scope of our multi-omics data and addresses your concern.

In parallel, we also strengthened the biological and translational interpretation of LAMC2 and IDO1 in the revised manuscript. First, we performed additional validation using three independent public cervical cancer transcriptomic datasets containing both invasive cervical cancer and normal cervical tissue samples (GSE63514, GSE9750, and GSE39001). LAMC2 was significantly upregulated in all three independent cohorts, whereas IDO1 was significantly upregulated in GSE9750 and GSE39001 and showed an increased but non-significant trend in GSE63514 (S6E Fig). These data have now been added as an independent transcriptomic validation analysis with boxplots and summary statistics.

Second, to further evaluate whether these candidates are functionally related to cervical cancer biology rather than merely being differentially expressed genes, we performed an exploratory in-silico perturbation analysis using the public single-cell RNA-seq dataset GSE168652, which contains cervical tumor tissue and matched adjacent normal tissue (**S6F-S6I Fig**). This analysis was used as exploratory single-cell-level functional support rather than as a clinical validation cohort. Virtual knockout of LAMC2 and IDO1 induced marked downstream changes in genes related to immune infiltration, epithelial differentiation, extracellular matrix remodeling, focal adhesion, inflammatory signaling, and tumor microenvironment remodeling. For LAMC2, the top affected genes included PTPRC, MCAM, RGS1, S100A8, CD53, SERPINB3, LUM, TYROBP, C1QB, CTSS, IFNG, and CD37, with pathway enrichment involving ECM-receptor interaction (**S6F-S6G Fig**). For IDO1, the top affected genes included KRT6A, SFN, PERP, S100A2, S100A9, IFI27, LY6D, SLPI, SERPINB3, SPARCL1, KRT17, KRT19, and SDC1, with pathway enrichment involving ECM-receptor interaction, focal adhesion, and regulation of the actin cytoskeleton (**S6H-S6I Fig**). Notably, several downstream genes, including S100A8 and SERPINB3, have previously been linked to cervical squamous cell carcinoma progression or prognosis in prior multi-omic studies. Together, these data support a biologically meaningful tissue-level role for the LAMC2/IDO1-associated marker program.

Finally, we have also revised the manuscript to clarify the intended clinical use case of these molecules. Specifically, LAMC2 may reflect extracellular matrix remodeling, epithelial-mesenchymal transition, and invasive tumor biology, whereas IDO1 may be more relevant to immune regulation, immune microenvironment classification, and immunotherapy-oriented stratification.

***Page 10:***

Stratification by LAMC2 expression revealed significant multi-omics alterations in the high-expression group, with upregulated genes enriched in key oncogenic pathways such as focal adhesion, PI3K-AKT, HPV infection, and ECM-receptor interaction (**Fig 5I**). These findings emphasize a LAMC2-associated ECM-receptor interaction model involving laminin-332 components, integrin receptor axes, focal adhesion kinases/scaffold proteins, and downstream PI3K-AKT and MAPK/EMT-related signaling outputs (**Fig 5J**).

***Page 37:***

**Fig. 5J**. Multi-omics model of LAMC2-associated ECM-receptor interaction in cervical cancer.

*In the Discussion, the authors state that "Persistent infection with high-risk human papillomavirus (HPV) induces elevated expression of programmed death protein-1 (PD-1) and its ligand (PD-L1) in cervical cancer." This requires refinement and further explanation - firstly, the PD1 expression is presumably on tumour infiltrating T-cells as a result of activation (and potentially subsequent exhaustion), whereas PD-L1 could be expressed on tumour cells and/or other cell types in the TME, including myeloid immune cells. It is not clear how (or indeed whether) these events are linked to persistent HPV infection as opposed to simply tumour progression, and the reference cited does not mention either HPV or cervical cancer.*

**Reply:** Thanks for this important and mechanistically precise comment. To address this issue, we have revised the Discussion to avoid stating that persistent HPV infection directly induces PD-1 and PD-L1 expression. Instead, we now describe increased PDCD1 and CD274 expression as features of an immune-inflamed and checkpoint-regulated tumor microenvironment. We also clarify that our bulk multi-omics data do not allow definitive assignment of CD274 expression to tumor cells versus immune or stromal cells, nor do they establish whether checkpoint expression is driven directly by persistent HPV infection, chronic antigen exposure, interferon-associated inflammation, or tumor progression.

We have also replaced the previously cited reference with cervical cancer- and HPV-relevant references. Specifically, we now cite articles (*Fan et al., Nature Genetics, 2023* and *Ou et al., Advanced Science, 2022*), both of which use single-cell and/or spatial multi-omics approaches to characterize the immune microenvironment of cervical squamous cell carcinoma. These studies provide a more appropriate basis for discussing the cell-type-aware interpretation of PDCD1 and CD274 expression in cervical cancer.

***Page 14:***

HPV-associated cervical cancer develops within a complex antigenic and inflammatory tumor microenvironment. In our cohort, immune-hot tumors showed increased expression of checkpoint-related molecules, including PDCD1, CD274, CTLA4, IDO1, and LAG3, together with cytotoxic markers such as GZMA, GZMB, and PRF1. PDCD1 expression likely reflects activated and/or exhausted tumor-infiltrating T cells, whereas CD274 expression may originate from malignant epithelial cells as well as non-malignant components of the tumor microenvironment, including myeloid-lineage cells and antigen-presenting cells. Therefore, we interpret the co-elevation of PDCD1 and CD274 as a feature of an immune-inflamed and checkpoint-regulated microenvironment, rather than as direct evidence that persistent HPV infection alone induces PD-1/PD-L1 expression (**S7I Fig**). Consistent with this phenotype, our analysis demonstrated that the immune-hot subgroup exhibits a significantly more favorable prognosis (**Fig 6H**) and lower Tumor Immune Dysfunction and Exclusion (TIDE) scores, suggesting a potentially better response to immune checkpoint inhibitors (ICIs) (**Fig 6I**).

***Page 9 of supporting information:***

**S7I Fig**. Correlation of PDCD1/CD274 with immune marker genes.

There are a lot of complex figure panels packed into the figures and as a result, they are often difficult to read. Particular examples include Figures 1F, 3C and 6A. The authors should carefully consider how they might simplify their figures and possibly move some content from main figures into supplementary. Sometimes less is more.

**Reply:** Thanks for your suggestion. We have accepted the suggestion and corrected these issues according your suggestions. The manuscript has been greatly improved by addressing your valuable comments.
