# Supplementary material for: Integrated multi-omics analysis reveals the molecular landscape and therapeutic strategies in Chinese cervical cancer patients
Source: PLoS Biol. 2026 Oct 5;24(10):e3004029. doi: 10.1371/journal.pbio.3004029 (PMC13637895; doi:10.1371/journal.pbio.3004029)
Supplement: Peer review history

# Editor report: Decision Letter 0

**DOI:** 10.1371/journal.pbio.3004029.r001

**Authors:** Richard Hodge

5 Feb 2026

Dear Dr Pan,

Thank you for submitting your manuscript entitled "Integration of multi-omics data to unveil the molecular landscape and therapeutic strategies in cervical cancer" for consideration as a Research Article by PLOS Biology. Please accept my sincere apologies for the delay in getting back to you with feedback.

Your manuscript has now been evaluated by the PLOS Biology editorial staff and I am writing to let you know that we would like to send your submission out for external peer review.

However, before we can send your manuscript to reviewers, we need you to complete your submission by providing the metadata that is required for full assessment. To this end, please login to Editorial Manager where you will find the paper in the 'Submissions Needing Revisions' folder on your homepage. Please click 'Revise Submission' from the Action Links and complete all additional questions in the submission questionnaire.

Once your full submission is complete, your paper will undergo a series of checks in preparation for peer review. After your manuscript has passed the checks it will be sent out for review. To provide the metadata for your submission, please Login to Editorial Manager (https://www.editorialmanager.com/pbiology) within two working days, i.e. by Feb 07 2026 11:59PM.

If your manuscript has been previously peer-reviewed at another journal, PLOS Biology is willing to work with those reviews in order to avoid re-starting the process. Submission of the previous reviews is entirely optional and our ability to use them effectively will depend on the willingness of the previous journal to confirm the content of the reports and share the reviewer identities. Please note that we reserve the right to invite additional reviewers if we consider that additional/independent reviewers are needed, although we aim to avoid this as far as possible. In our experience, working with previous reviews does save time.

If you would like us to consider previous reviewer reports, please edit your cover letter to let us know and include the name of the journal where the work was previously considered and the manuscript ID it was given. In addition, please upload a response to the reviews as a 'Prior Peer Review' file type, which should include the reports in full and a point-by-point reply detailing how you have or plan to address the reviewers' concerns.

During the process of completing your manuscript submission, you will be invited to opt-in to posting your pre-review manuscript as a bioRxiv preprint. Visit http://journals.plos.org/plosbiology/s/preprints for full details. If you consent to posting your current manuscript as a preprint, please upload a single Preprint PDF.

Feel free to email us at plosbiology@plos.org if you have any queries relating to your submission.

Kind regards,

Richard

Richard Hodge, PhD

Senior Editor, PLOS Biology

rhodge@plos.org

PLOS

Empowering researchers to transform science

Carlyle House, Carlyle Road, Cambridge, CB4 3DN, United Kingdom

California (U.S.) corporation #C2354500, based in San Francisco

# Peer review: Decision Letter 1

**DOI:** 10.1371/journal.pbio.3004029.r002

**Authors:** Richard Hodge

5 May 2026

Dear Dr Pan,

Thank you for your patience while your manuscript "Integration of multi-omics data to unveil the molecular landscape and therapeutic strategies in cervical cancer" was peer-reviewed at PLOS Biology. Please accept my sincere apologies for the delays that you have experienced during the peer review process. Your manuscript has now been evaluated by the PLOS Biology editors, an Academic Editor with relevant expertise, and by two independent reviewers.

In light of the reviews, which you will find at the end of this email, we would like to invite you to revise the work to thoroughly address the reviewers' reports.

As you will see, the reviewers are generally positive about the study, although they each raise overlapping and important concerns that the manuscript lacks important methodological/reporting details and quality control metrics. In addition, Reviewer #1 raises concerns with over-interpretation of the results given the lack of validation in an independent validation cohort and asks that additional analyses are provided to support the claims. Finally, Reviewer #2 notes that he/she has some concerns with the methodology used which requires further clarification, and with the organisation/presentation of the manuscript.

Given the extent of revision needed, we cannot make a decision about publication until we have seen the revised manuscript and your response to the reviewers' comments. Your revised manuscript is likely to be sent for further evaluation by all or a subset of the reviewers.

In addition to these revisions, you will need to complete some formatting changes, which you will receive in a follow up email. A member of our team will be in touch with a set of requests shortly.

We expect to receive your revised manuscript within 3 months. Please email us (plosbiology@plos.org) if you have any questions or concerns, or would like to request an extension.

At this stage, your manuscript remains formally under active consideration at our journal; please notify us by email if you do not intend to submit a revision so that we may withdraw it.

**IMPORTANT - SUBMITTING YOUR REVISION**

Your revisions should address the specific points made by each reviewer. Please submit the following files along with your revised manuscript:

1. A 'Response to Reviewers' file - this should detail your responses to the editorial requests, present a point-by-point response to all of the reviewers' comments, and indicate the changes made to the manuscript.

*NOTE: In your point-by-point response to the reviewers, please provide the full context of each review. Do not selectively quote paragraphs or sentences to reply to. The entire set of reviewer comments should be present in full and each specific point should be responded to individually, point by point.

You should also cite any additional relevant literature that has been published since the original submission and mention any additional citations in your response.

2. In addition to a clean copy of the manuscript, please also upload a 'track-changes' version of your manuscript that specifies the edits made. This should be uploaded as a "Revised Article with Changes Highlighted" file type.

*Re-submission Checklist*

When you are ready to resubmit your revised manuscript, please refer to this re-submission checklist: https://plos.io/Biology_Checklist

To submit a revised version of your manuscript, please go to https://www.editorialmanager.com/pbiology/ and log in as an Author. Click the link labelled 'Submissions Needing Revision' where you will find your submission record.

Please make sure to read the following important policies and guidelines while preparing your revision:

*Published Peer Review*

Please note while forming your response, if your article is accepted, you may have the opportunity to make the peer review history publicly available. The record will include editor decision letters (with reviews) and your responses to reviewer comments. If eligible, we will contact you to opt in or out. Please see here for more details:

https://blogs.plos.org/plos/2019/05/plos-journals-now-open-for-published-peer-review/

*PLOS Data Policy*

Please note that as a condition of publication PLOS' data policy (http://journals.plos.org/plosbiology/s/data-availability) requires that you make available all data used to draw the conclusions arrived at in your manuscript. If you have not already done so, you must include any data used in your manuscript either in appropriate repositories, within the body of the manuscript, or as supporting information (N.B. this includes any numerical values that were used to generate graphs, histograms etc.). For an example see here: http://www.plosbiology.org/article/info%3Adoi%2F10.1371%2Fjournal.pbio.1001908#s5

*Blot and Gel Data Policy*

We require the original, uncropped and minimally adjusted images supporting all blot and gel results reported in an article's figures or Supporting Information files. We will require these files before a manuscript can be accepted so please prepare them now, if you have not already uploaded them. Please carefully read our guidelines for how to prepare and upload this data: https://journals.plos.org/plosbiology/s/figures#loc-blot-and-gel-reporting-requirements

*Protocols deposition*

To enhance the reproducibility of your results, we recommend that if applicable you deposit your laboratory protocols in protocols.io, where a protocol can be assigned its own identifier (DOI) such that it can be cited independently in the future. Additionally, PLOS ONE offers an option for publishing peer-reviewed Lab Protocol articles, which describe protocols hosted on protocols.io. Read more information on sharing protocols at https://plos.org/protocols?utm_medium=editorial-email&utm_source=authorletters&utm_campaign=protocols

Thank you again for your submission to our journal. We hope that our editorial process has been constructive thus far, and we welcome your feedback at any time. Please don't hesitate to contact us if you have any questions or comments.

Best regards,

Richard

Richard Hodge, PhD

Senior Editor, PLOS Biology

rhodge@plos.org

------------------------------------

REVIEWS:

Reviewer #1: The manuscript 'Integration of multi-omics data to unveil the molecular landscape and therapeutic strategies in cervical cancer' by Pan et al is a technically sound, high quality compendium of DNA, RNA, protein, and phosphoprotein data from a cohort of 112 Chinese patients with cervical cancer (CC), along with paired 'normal adjacent tissue' (NAT) for most of the patients. Appropriate ethical issues including informed consent are satisfactory. Strengths include a cohort of Chinese / East Asian patients of appropriate size to complement existing studies of predominantly white / North American women, the ability to compare directly to paired NAT from the same subject, and the technical quality of the DNA (WES), RNA (RNA-Seq), protein (TMT LC-MS/MS) and phosphoprotein (IMAC enriched MS/MS) data. The data appear to be appropriately available, although with different authors. It is clear that the multiomic data generated for this study represents a useful resource for cancer researchers and is likely to be mined by multiple groups for bioinformatic insights.

That said, there are several substantial issues that need to be addressed before these data can be used by others as a trusted resource.

1.It is unclear who did the pathological review of the tumor specimens. The authors merely state that 'We also collected the clinical parameters of the patients, including age, tumor grade and stage (Fig 1B and S1 Table)'. Ideally, in a study of this nature, a single pathologist or team of pathologists reviews all samples to insure that there is agreement about the diagnosis of the tumor. The Supplemental Table 1 contains multiple columns labelled 'Stage' or 'Grade', and it is not clear which column represents the final consensus.

2.The Methods section is missing some key information, specifically whether the tumor tissues were subjected to either macro or micro dissection, and what the quality control criteria were for per cent cellularity, per cent tumor tissue, and per cent necrosis. Additionally, the description of protein processing for MS analysis does not specify how much tissue mass was used per extraction. These are important quality control metrics that are essential for replication of the results

3.There is no description of the procedure for obtaining NAT. How far away was the NAT from the tumor tissue? Were slides of the NAT reviewed by a pathologist to insure that the tissues were, in fact, of normal histology? Ideally, photomicrographs of representative CC and NAT stained with hematoxylin and eosin would be provided.

Secondly, there is the usual issue with over-interpretation of results in this type of proteogenomic study, exacerbated by the lack of validation either in an independent patient cohort or in an experimental system. While these issues are usually resolved over time as other investigators build upon this work, the authors of the current study need to be transparent about the limitations of the current study, and the types of further work that would be necessary to support their claims. Following is a sample of the most problematic claims that need to be qualified.

1.The unsupervised hierarchical heat maps in Figure 4A do not really support the idea of 3 subtypes at the RNA, protein, and phosphoprotein levels, independent of HPV integration status. A look at the top ribbon indicates that the three subgroups are almost entirely driven by HPV integration status (non-integrated, silent, or productive) with a few discrepancies. It would have been far more interesting to do a supervised clustering based on HPV integration status and compare the resulting heatmaps.

2.The claims for modification of overall survival by PLOD2 status and TFRC status are over-simplified, if not outright overstated. For PLOD2, survival past 3000 days is indistinguishable for PLOD2 high versus low. For TFRC, the survival up to 2000 days is identical for TFRC high versus low. There is no acknowledgement of the clinical consequences of these survival patterns, and the overall p values are only marginally significant.

3.While the levels of LAMC2 and IDO1 are clearly very different between CC and NAT, it is not clear how these would be clinically useful as biomarkers, given the lack of validation in an independent cohort, and the absence of any information regarding their appearance in serum or plasma. More thought needs to be devoted to the clinical use case for candidate biomarkers.

4.The entire immune landscape section is very confusing due to the absence of key details. The authors claim 6 immune subtypes without providing a consensus clustering matrix (as in Figure S5) to justify 6 distinct subtypes, and they never really describe what is driving the 6 subtypes. They immediately go to lumping the 6 subtypes into 3: 'immune hot, immune warm, and immune cold', and all subsequent comparisons are between hot, warm, and cold. The bar graphs in 6D, 6E, and 6G appear to support 3 subtypes, not 6, and the K-M plot in Figure 6H shows a very clear difference in survival which warrants further analysis and experimentation. However, the rather arbitrary 6 immune subtypes merely confuses the analysis.

Minor typo in the legend for Figure 5; between is missing the 'b' [etween].

Reviewer #2: This manuscript describes the multi-omics analysis of a Chinese cervical cancer cohort, which complements notable existing studies from European, African and US-based cohorts and therefore has the potential to make an important contribution to our understanding of cervical cancer biology and its similarities across different ethnic groups. A particular strength of this study, which sets it apart from the aforementioned studies is the application of global proteomics to interrogate differential protein expression and activation states on a much larger (and unbiased) scale than the RPPA approach used by TCGA, for example. I do have some concerns the regarding clarity of reporting and the methodology employed.

The study relies heavily on comparisons between three subgroups defined by the authors based on the physical status of the HPV genome and on HPV transcriptional activity: HPV-integrated with evidence of HPV gene expression ("productive"); HPV-integrated without evidence of HPV gene expression ("silent") and non-integrated subgroups. However, insufficient detail is provided in the methods for the reader to understand how these groups were defined and to give confidence that samples have been accurately attributed to these groups. The methods section lacks any description of how HPV typing and assessment of HPV gene expression was conducted. In the description of alignment for the WES and RNA-seq data, only alignment to the human reference genome is mentioned but presumably, HPV genomes were also included, either appended to GRCh38 to make a combined human/viral reference, or unaligned reads from the BAM file generated by alignment to GRCh38 were taken and aligned to HPV genomes? If not, how were samples classified as transcriptionally active or silent, and where does the evidence supporting integrated versus non-integrated status for each sample come from? This needs to be detailed for both WES and RNA-seq data (including information on the source of the HPV genomes, accession numbers etc). Note that whole exome sequencing is not a reliable method (at least when used in isolation) by which to determine HPV integration status. Whole genome sequencing (data now available for TCGA's cohort, so deriving accurate integration status for TCGA samples from this could be of use to the authors) is much more reliable for detecting and mapping HPV integration. In lieu of this, capturing HPV genome sequences in addition to the human exome would enable more reliable detection of integration than relying on exome data alone.

Related to the above point, it is surprising to see so many cervical SCCs in this cohort in which HPV gene expression appears to have been undetectable using RNA sequencing. This is a significant difference from TCGA's cohort, from the cohort investigated by Ojesina et al (cited as reference 20) and from CGCI's Uganda cohort (https://doi.org/10.1038/s41588-020-0673-7). In the absence of any information in the methods section about how HPV reads were identified and quantified, it is impossible for me to assess what may explain this unexpected finding. Was a cutoff for HPV transcript reads defined, below which a sample was defined as silent?

The schematic diagram in Figure 1A is helpful in explaining how many cancer samples were analysed in each of the different experiments (112 for proteomics, and subsets of 97 for RNA-seq (+ 97 NAT) and 98 for whole exome seq (+81 NAT). But the text does not correspond very clearly to this, so for example, in the Results section "Thus, we conducted genomic analysis on paired tumors and adjacent tissues from 98 CC patients" Should this not read "paired tumors and adjacent tissues from 81 CC patients, and tumor-only from an additional 17?" If so, it is also essential to clearly describe how somatic variants were called from the 17 samples for which no paired normal tissue was taken. Or was DNA isolated from blood, or NAT from the proteomics study (for which NAT was available for all 112 cases) to get the germline exome sequence data?

Why are the sample numbers different between Figure 1G (n=98) and Figure 1H (n=81)? Why could HRD scores not be defined for the entire WES cohort? Again, essential methodological detail is missing - there is no description of how HRD scores were calculated in the methods section.

Mutational signature analysis - although the authors state in the methods that "The number of signatures was determined by choosing the reflection point in the curves of explained variance and residual sum of squares (RSS)", which is a reasonable approach, it looks like they have overestimated the number of signatures present. For example their signature S5 clearly looks to be a combination of S1 and S3 (SBS2 and SBS13 respectively), rather than being a separate signature. It would be worth looking again at this analysis and potentially restricting to signatures that have been reported to be present in cervical cancer (e.g. by reference to COSMIC (https://cancer.sanger.ac.uk/signatures/) and Signal (https://signal.mutationalsignatures.com/).

The introduction is very brief and while TCGA's 2017 landscape study is mentioned, it does not provide a strong overview of the context in which the present manuscript should be viewed. Key cervical cancer genomics studies such as Ojesina et al Nature 2014 (which preceded TCGA's 2017 publication) should at least be discussed at this point. The decision to highlight SHKBP1, ERBB3, and TGFBR2 from TCGA's publication is also surprising, given that these are not among the most common somatic alterations detected in that study, or in the previous study from Ojesina et al. Driver mutations in PIK3CA and EP300 for example, were reported at much higher frequencies in both these studies. The multi-omics analysis of cervical squamous cell carcinoma conducted by Chakravarthy et al (Chakravarthy et al Nat Comms 2022 (https://doi.org/10.1038/s41467-022-33544-x) is also highly relevant to the work conducted here and made observations that are relevant to this study e.g. concerning EMT, Hippo/YAP signalling and the immune microenvironment. The authors should make more effort to consider their findings in the context of these previous studies and to highlight similarities and differences. This is particularly important given the fact that as they correctly point out, Asian patients have been underrepresented in these studies, so it would be very useful to understand whether there are potential differences by ethnicity. The authors might consider for example, allocating their samples to the gene expression-based clusters identified by TCGA and by Chakravarthy et al. Given that these clusters were associated with differences in survival, it would be very interesting to see whether these differences are apparent in this cohort of Asian patients. Finally, given the rationale for identifying markers that could predict ICI response, it would also be useful to include some background context on the use of ICI in cervical cancer.

Initial description of the cohort: I notice from Table S1 that all tumours analysed are squamous cell carcinomas - it would be useful to state this, either in the introduction or at the beginning of the results section.

I would advise against use of the term 'productive' to describe tumours in which HPV gene expression is detected, as this will cause confusion with the term productive HPV infection - used to describe the normal life cycle of the virus which results in the production of HPV virions. A suitable alternative might be 'transcriptionally active', or simply 'active'.

For the statement, "PIK3CA mutations were predominantly localized to the helical domain (E542K, E545K), a pattern consistent with bladder cancer and HPV-positive head and neck squamous cell carcinomas, but distinct from breast cancer (Fig 1E). These substitutions are potentially linked to APOBEC-mediated mutagenesis…." The correct primary reference to cite for this is Henderson et al Cell Reports 2014 (https://doi.org/10.1016/j.celrep.2014.05.012).

Since Figure 5J represents findings from protein-level data, protein names should be used (i.e. PIK3CA encodes the p110-alpha catalytic subunit of PI3K - is this the protein that the authors have found to be phosphorylated, as suggested in the schematic diagram? Phosphorylation of p110-alpha is not a well described regulatory mechanism, rather it is recruited to activated RTKs via binding of its regulatory subunit (p85) to phosphotyrosine motifs on the receptors. If the phospho-proteomics data do not identify PI3K phosphorylation but rather an inferred increase in activation based on increased AKT phosphorylation, the diagram should be modified accordingly.

In the Discussion, the authors state that "Persistent infection with high-risk human papillomavirus (HPV) induces elevated expression of programmed death protein-1 (PD-1) and its ligand (PD-L1) in cervical cancer." This requires refinement and further explanation - firstly, the PD1 expression is presumably on tumour infiltrating T-cells as a result of activation (and potentially subsequent exhaustion), whereas PD-L1 could be expressed on tumour cells and/or other cell types in the TME, including myeloid immune cells. It is not clear how (or indeed whether) these events are linked to persistent HPV infection as opposed to simply tumour progression, and the reference cited does not mention either HPV or cervical cancer.

There are a lot of complex figure panels packed into the figures and as a result, they are often difficult to read. Particular examples include Figures 1F, 3C and 6A. The authors should carefully consider how they might simplify their figures and possibly move some content from main figures into supplementary. Sometimes less is more.

# Author comment: Author response to Decision Letter 2

**DOI:** 10.1371/journal.pbio.3004029.r003

3 Aug 2026

Attachment Submitted filename: Response to Reviewers.docx

# Peer review: Decision Letter 2

**DOI:** 10.1371/journal.pbio.3004029.r004

**Authors:** Richard Hodge

4 Sep 2026

Dear Dr Pan,

Thank you for your patience while we considered your revised manuscript "Integration of multi-omics data to unveil the molecular landscape and therapeutic strategies in cervical cancer" for publication as a Research Article at PLOS Biology. Please accept my apologies for the delays that you have experienced during this round of the peer review process. This revised version of your manuscript has been evaluated by the PLOS Biology editors, the Academic Editor and the original reviewers .

Based on the reviews, we are likely to accept this manuscript for publication, provided you satisfactorily address the remaining points raised by Reviewer #1. Please also make sure to address the following data and other policy-related requests that I have provided below (A-G):

(A) We routinely suggest changes to titles to ensure maximum accessibility for a broad, non-specialist readership. In this case, we suggest a minor edit to the title, as follows, to make it clear that the study is focused on Chinese populations. Please ensure you change both the manuscript file and the online submission system, as they need to match for final acceptance:

“Integrated multi-omics analysis reveals the molecular landscape and therapeutic strategies in Chinese cervical cancer patients”

(B) After discussion within the editorial team, we think that your manuscript would be a better fit as a 'Methods and Resources Article' at the journal (in the Resource category rather than Method). I have therefore changed the article type during this round of revision, but please do let me know if you have any questions or concerns about this.

(C) You may be aware of the PLOS Data Policy, which requires that all data be made available without restriction: http://journals.plos.org/plosbiology/s/data-availability. For more information, please also see this editorial: http://dx.doi.org/10.1371/journal.pbio.1001797

Note that we do not require all raw data. Rather, we ask that all individual quantitative observations that underlie the data summarized in the figures and results of your paper be made available in one of the following forms:

-Supplementary files (e.g., excel). Please ensure that all data files are uploaded as 'Supporting Information' and are invariably referred to (in the manuscript, figure legends, and the Description field when uploading your files) using the following format verbatim: S1 Data, S2 Data, etc. Multiple panels of a single or even several figures can be included as multiple sheets in one excel file that is saved using exactly the following convention: S1_Data.xlsx (using an underscore).

-Deposition in a publicly available repository. Please also provide the accession code or a reviewer link so that we may view your data before publication.

Regardless of the method selected, please ensure that you provide the individual numerical values that underlie the summary data displayed in the following figure panels as they are essential for readers to assess your analysis and to reproduce it:

Figure 1C-D, 1G-H, 2A-B, 2D, 2F-I, 3B-D, 3G, 3J-M, 4B-D, 4F-H, 5B-G, 5I, 6A-O, S1B, S1D-J, S2A-I, S3A-L , S4A-C, S5A-C, S6A-I, S7A-I

NOTE: the numerical data provided should include all replicates AND the way in which the plotted mean and errors were derived (it should not present only the mean/average values).

(D) Thank you for providing the WES, RNA-seq, proteomic, and phosphoproteomic datasets in Github (https://github.com/ComputationalEpigeneticsLab/Chinese-CC-MOCC-v1.0.0). However, Please note that we cannot accept sole deposition of data in GitHub, as this could be changed after publication. However, you can archive this version of your publicly available GitHub code to Zenodo. Once you do this, it will generate a DOI number, which you will need to provide in the Data Accessibility Statement (you are welcome to also provide the GitHub access information). See the process for doing this here: https://docs.github.com/en/repositories/archiving-a-github-repository/referencing-and-citing-content

(E) Please also ensure that each of the relevant figure legends in your manuscript include information on *WHERE THE UNDERLYING DATA CAN BE FOUND*, and ensure your supplemental data file/s has a legend.

(F) Please ensure that your Data Statement in the submission system accurately describes where your data can be found and is in final format, as it will be published as written there. In particular, I note that the statement states that the WES, RNA-seq, proteomic, and phosphoproteomic data were downloaded from Github, but I think the correct term here should be ‘deposited in’. In addition, could you please confirm whether the data deposited in Github are the raw datasets but anonymized due to patient confidentiality?

(G) Per journal policy, if you have generated any custom code during the course of this investigation, please make it available without restrictions. Please ensure that the code is sufficiently well documented and reusable, and that your Data Statement in the Editorial Manager submission system accurately describes where your code can be found. More information on our Code Policy, what and how to share can be found here: https://journals.plos.org/plosbiology/s/code-availability

Like before, please note that we cannot accept sole deposition of code in GitHub, as this could be changed after publication. However, you can archive this version of your publicly available GitHub code to Zenodo. Once you do this, it will generate a DOI number, which you will need to provide in the Data Accessibility Statement (you are welcome to also provide the GitHub access information). See the process for doing this here: https://docs.github.com/en/repositories/archiving-a-github-repository/referencing-and-citing-content

------------------------------------------------------------------------

As you address these items, please take this last chance to review your reference list to ensure that it is complete and correct. If you have cited papers that have been retracted, please include the rationale for doing so in the manuscript text, or remove these references and replace them with relevant current references. Any changes to the reference list should be mentioned in the cover letter that accompanies your revised manuscript.

In addition to these revisions, you may need to complete some formatting changes, which you will receive in a follow up email. A member of our team will be in touch with a set of requests shortly. If you do not receive a separate email within a few days, please assume that checks have been completed, and no additional changes are required.

We expect to receive your revised manuscript within two weeks.

To submit your revision, please go to https://www.editorialmanager.com/pbiology/ and log in as an Author. Click the link labelled 'Submissions Needing Revision' to find your submission record. Your revised submission must include the following:

- a cover letter that should detail your responses to any editorial requests, if applicable, and whether changes have been made to the reference list

- a Response to Reviewers file that provides a detailed response to the reviewers' comments (if applicable, if not applicable please do not delete your existing 'Response to Reviewers' file.)

- a track-changes file indicating any changes that you have made to the manuscript.

NOTE: If Supporting Information files are included with your article, note that these are not copyedited and will be published as they are submitted. Please ensure that these files are legible and of high quality (at least 300 dpi) in an easily accessible file format. For this reason, please be aware that any references listed in an SI file will not be indexed. For more information, see our Supporting Information guidelines:

https://journals.plos.org/plosbiology/s/supporting-information

*Published Peer Review History*

Please note that you may have the opportunity to make the peer review history publicly available. The record will include editor decision letters (with reviews) and your responses to reviewer comments. If eligible, we will contact you to opt in or out. Please see here for more details:

https://plos.org/published-peer-review-history/

*Press*

Should you, your institution's press office or the journal office choose to press release your paper, please ensure you have opted out of Early Article Posting on the submission form. We ask that you notify us as soon as possible if you or your institution is planning to press release the article.

*Protocols deposition*

To enhance the reproducibility of your results, we recommend that if applicable you deposit your laboratory protocols in protocols.io, where a protocol can be assigned its own identifier (DOI) such that it can be cited independently in the future. Additionally, PLOS ONE offers an option for publishing peer-reviewed Lab Protocol articles, which describe protocols hosted on protocols.io. Read more information on sharing protocols at https://plos.org/protocols?utm_medium=editorial-email&utm_source=authorletters&utm_campaign=protocols

Please do not hesitate to contact me should you have any questions.

Best wishes,

Richard

Richard Hodge, PhD

Senior Editor, PLOS Biology

rhodge@plos.org

------------------------------------------------------------------------

Reviewer remarks:

Reviewer #1: The authors have done an admirable of correcting the over-interpretations in the original submission, most usually by qualifying potential inferences as requiring further clinical validation in independent cohorts. They have also clarified that the current emphasis is on understanding biological mechanisms rather than identifying and validating therapeutic targets. They have also added important information about the pathological review of the samples, and the criteria for acceptable cancer and normal adjacent tissues.

The one further revision that would substantially improve the manuscript is to call out explicitly, in the Discussion, how the results in this multi-omic profiling of Chinese patients differed from prior work done in mostly North American - European populations. From reading the manuscript, it appears most results were concordant between TCGA and the current study, and that is a perfectly acceptable answer that does not detract from the importance of the current work. However, since a major justification for the current study is the under-study of Asian populations, the Discussion is obligated to point out ways in which the Chinese population resembles other studies, and ways in which the Chinese population appears to be unique.

Reviewer #2 (Tim Fenton, identifies himself): The authors have made a good job of carefully addressing my previous concerns, particularly through clarification of the methods and description of the cohort. I find the revised manuscript to be much easier to follow and believe it makes an important contribution to our understanding of cervical cancer.

# Author comment: Author response to Decision Letter 3

**DOI:** 10.1371/journal.pbio.3004029.r005

14 Sep 2026

Attachment Submitted filename: Response_to_Reviewers_auresp_3.docx

# Editor report: Decision Letter 3

**DOI:** 10.1371/journal.pbio.3004029.r006

**Authors:** Richard Hodge

15 Sep 2026

Dear Dr Pan,

On behalf of my colleagues and the Academic Editor, Elena Rainero, I am pleased to say that we can accept your manuscript for publication, provided you address any remaining formatting and reporting issues. These will be detailed in an email you should receive within 2-3 business days from our colleagues in the journal operations team; no action is required from you until then. Please note that we will not be able to formally accept your manuscript and schedule it for publication until you have completed any requested changes.

Please take a minute to log into Editorial Manager at http://www.editorialmanager.com/pbiology/, click the "Update My Information" link at the top of the page, and update your user information to ensure an efficient production process.

PRESS

We frequently collaborate with press offices. If your institution or institutions have a press office, please notify them about your upcoming paper at this point, to enable them to help maximise its impact. If the press office is planning to promote your findings, we would be grateful if they could coordinate with biologypress@plos.org. If you have previously opted in to the early version process, we ask that you notify us immediately of any press plans so that we may opt out on your behalf.

We also ask that you take this opportunity to read our Embargo Policy regarding the discussion, promotion and media coverage of work that is yet to be published by PLOS. As your manuscript is not yet published, it is bound by the conditions of our Embargo Policy. Please be aware that this policy is in place both to ensure that any press coverage of your article is fully substantiated and to provide a direct link between such coverage and the published work. For full details of our Embargo Policy, please visit http://www.plos.org/about/media-inquiries/embargo-policy/.

Thank you again for choosing PLOS Biology for publication and supporting Open Access publishing. We look forward to publishing your study.

Best wishes,

Richard

Richard Hodge, PhD

Senior Editor, PLOS Biology

rhodge@plos.org

PLOS

Empowering researchers to transform science

Carlyle House, Carlyle Road, Cambridge, CB4 3DN, United Kingdom

California (U.S.) corporation #C2354500, based in San Francisco
